# Aβ accumulation causes MVB enlargement and is modelled by dominant negative VPS4A

**DOI:** 10.1186/s13024-017-0203-y

**Published:** 2017-08-23

**Authors:** Katarina Willén, James R. Edgar, Takafumi Hasegawa, Nobuyuki Tanaka, Clare E. Futter, Gunnar K. Gouras

**Affiliations:** 1 Department of Experimental Medical Science, Lund University, 221 84 Lund, Sweden; 2 Cambridge Institute for Medical Research, University of Cambridge, Cambridge, CB2 0XY UK; 3 UCL Institute of Ophthalmology, London, EC1V 9EL UK; 4 Division of Neurology, Department of Neuroscience and Sensory Organs, Tohoku University Graduate School of Medicine, Sendai, 980-8574 Japan; 5 Division of Cancer Biology and Therapeutics, Miyagi Cancer Center Research Institute, Natori, 981-1293 Japan

**Keywords:** Alzheimer’s disease, Amyloid, Endocytosis, Multivesicular body, Tau

## Abstract

**Background:**

Alzheimer’s disease (AD)-linked β-amyloid (Aβ) accumulates in multivesicular bodies (MVBs) with the onset of AD pathogenesis. Alterations in endosomes are among the earliest changes associated with AD but the mechanism(s) that cause endosome enlargement and the effects of MVB dysfunction on Aβ accumulation and tau pathology are incompletely understood.

**Methods:**

MVB size and Aβ fibrils in primary neurons were visualized by electron microscopy and confocal fluorescent microscopy. MVB-dysfunction, modelled by expression of dominant negative VPS4A (dnVPS4A), was analysed by biochemical methods and exosome isolation.

**Results:**

Here we show that AD transgenic neurons have enlarged MVBs compared to wild type neurons. Uptake of exogenous Aβ also leads to enlarged MVBs in wild type neurons and generates fibril-like structures in endocytic vesicles. With time fibrillar oligomers/fibrils can extend out of the endocytic vesicles and are eventually detectable extracellularly. Further, endosomal sorting complexes required for transport (ESCRT) components were found associated with amyloid plaques in AD transgenic mice. The phenotypes previously reported in AD transgenic neurons, with net increased intracellular levels and reduced secretion of Aβ, were mimicked by blocking recycling of ESCRT-III by dnVPS4A. DnVPS4A further resembled AD pathology by increasing tau phosphorylation at serine 396 and increasing markers of autophagy.

**Conclusions:**

We demonstrate that Aβ leads to MVB enlargement and that amyloid fibres can form within the endocytic pathway of neurons. These results are consistent with the scenario of the endosome-lysosome system representing the site of initiation of Aβ aggregation. In turn, a dominant negative form of the CHMP2B-interacting protein VPS4A, which alters MVBs, leads to accumulation and aggregation of Aβ as well as tau phosphorylation, mimicking the cellular changes in AD.

**Electronic supplementary material:**

The online version of this article (doi:10.1186/s13024-017-0203-y) contains supplementary material, which is available to authorized users.

## Background

Alzheimer’s disease (AD) is characterized by progressive decline in cognitive function, anatomical selective loss of synapses and neurons, and aggregation of the β-amyloid peptide (Aβ) in amyloid plaques and hyperphosphorylated tau in neurofibrillary tangles (NFTs). Although plaques are extracellular aggregates of Aβ, accumulation of Aβ42, the most pathogenic Aβ peptide, begins within neurons in AD [1–3] and in AD transgenic mouse models [4–6]. In AD transgenic mice, cognitive, physiological and structural impairments appear prior to plaques [7–9] and are accompanied by intraneuronal Aβ peptide accumulation, supporting that accumulation of intraneuronal Aβ peptides is one of the earliest events in AD pathogenesis [10].

Abnormalities in the endocytic pathway are also among the earliest pathological features reported in AD, preceding the classical pathological markers of Aβ plaques and NFTs [11]. Specifically, enlargement of Rab5-positive early endosomes and Rab7-positive late endosomes were reported in AD [12, 13], as well as progressive accumulation of multivesicular bodies (MVBs), lysosomes and autophagic vacuoles [14]. The amyloidogenic cleavage of APP occurs predominantly in endosomes [15–19]. Proteins in the amyloidogenic pathway (APP, the β-site APP cleaving enzyme (BACE1) and the γ-secretase that generates the Aβ peptides) are transmembrane proteins that traffic through the secretory pathway as well as the endocytic pathway. Immuno-electron microscopy revealed that particularly the limiting membrane of MVBs are the normal location of Aβ42 in neurons of the brain and are the sites of Aβ accumulation during AD pathogenesis [20], especially at synapses [5]. Sorting of EGFR via the MVB pathway was impaired by endosomal Aβ accumulation in cultured AD transgenic neurons [21]. Translocation into MVBs appeared particularly affected, suggesting Aβ dependent dysfunction of the late endosomal sorting complexes required for transport (ESCRT) pathway in AD.

The ESCRTs are a set of proteins conserved from yeast to mammals that regulate and drive formation of the intraluminal vesicles of MVBs. They assemble into distinct subcomplexes: ESCRT-0, ESCRT-I, ESCRT-II and ESCRT-III. Their sequential action directs the sorting of ubiquitinated transmembrane proteins and the inward budding of intraluminal vesicles (ILVs) into the lumen of endosomes, thereby generating MVBs that then either deliver membrane-associated cargo to the lysosome for degradation, release the intraluminal vesicles (then called exosomes) via fusion with the plasma membrane or traffic cargo back to the Golgi apparatus. ESCRT-III subunits, among them CHMP2B, are inactive monomers in the cytoplasm [22] that assemble on endosomal membranes in an ordered manner to generate the transient ESCRT-III complex. CHMP2B, linked genetically to frontotemporal dementia (FTD) and AD [23, 24], directly interacts with and recruits the VPS4 AAA–ATPase complex that disassembles ESCRT-III, and genome-wide association studies for late onset AD identified an association with VPS4B [25].

Given the cumulative genetic, biological and pathological evidence implicating Aβ in AD, and the early accumulation of Aβ in MVBs in AD, we set out to test our first hypothesis (1) that Aβ can cause the abnormal endosomal phenotype seen in AD. To determine this, we investigated the effects of Aβ on MVB size and Aβ aggregation in late endosomes. Since Aβ accumulates particularly at the outer limiting membrane of MVBs where ESCRTs reside and since ESCRT dysfunction leads to endosomal enlargement we also tested our second hypothesis (2) that Aβ causes dysfunction of the ESCRT pathway. This was investigated by examining changes in ESCRT proteins in primary neurons, as well as modulating the late ESCRT pathway to examine how this influences Aβ accumulation.

Here we provide experimental evidence of Aβ-dependent MVB enlargement as well as Aβ aggregation within late endocytic compartments of neurons. Consistent with the scenario of MVBs representing the site of initiation of Aβ aggregation, the accumulation of neuronal ESCRT components was evident in amyloid plaques. Moreover, dysfunction of ESCRT-III, modelled by dominant negative VPS4A (dnVPS4A) mimicked the Aβ accumulation and aggregation in MVBs as well as the enlarged late endosomal size seen in AD. These results support a novel scenario where a vicious cycle of ESCRT-dependent late endosomal dysfunction causes further Aβ accumulation as well as AD-pathogenic tau phosphorylation.

## Methods

### Cell culture

Primary neuronal cultures were generated from B6.Cg-Tg (APPswe, PSEN1dE9)85Dbo/Mmjax mice (APP/PS1) AD transgenic (tg) and wild-type (wt) mouse embryos. The APP sequence in APP/PS1 encodes a chimeric mouse/human APP (Mo/HuAPP695swe) that was humanized by modifying three amino acids, and introducing the Swedish AD mutation. The PS1 sequence encodes human presenilin 1 lacking exon 9 (dE9) that models AD-associated mutations in PS1. Both APPswe and PS1 are independently controlled by the prion protein promoter. Primary neuronal cultures were prepared from cortices and hippocampi of embryonic day 15 embryos as previously described [9]. In brief, E15 brain tissue was dissociated by trypsinization and trituration in DMEM with 10% fetal bovine serum (Gibco). Dissociated neurons were cultured on poly-D-lysine (Sigma) coated plates or glass coverslips (Bellco Glass Inc.) and were maintained until 12 and 19 DIV in neurobasal medium (Gibco), B27 supplement (Gibco), glutamine (Invitrogen) and antibiotics (ThermoScientific).

Wild type mouse N2a neuroblastoma cells (N2a) or N2a cells stably transfected with the 670/671 Swedish mutation human APP (Swe) [26] or wild-type α-synuclein with HA-Tag (α-syn) were grown on 10 cm dishes or coverslips.

### Electron microscopy

Cells were grown on Thermanox coverslips (Nalgene, Nunc) and fixed with 2% PFA, 2.5% glutaraldehyde in 0.1 M cacodylate. Cells were then secondarily fixed with 1% osmium tetroxide followed by incubation with 1% tannic acid to enhance contrast. Cells were dehydrated using increasing percentages of ethanol before being embedded onto Epoxy resin (Agar scientific, UK) stubs. Coverslips were cured overnight at 65 °C. Ultrathin sections were cut using a diamond knife mounted to a Reichert ultracut S ultramicrotome and sections were collected onto copper grids. Grids were post-stained with drops of lead citrate. Sections were viewed on a FEI Tecnai transmission electron microscope (Eindhoven, The Netherlands) at a working voltage of 80 kV. BSA-gold was prepared as previously described [27]. For quantification of MVB diameter, MVBs were defined as organelles containing intraluminal vesicles and monomeric rather than flocculated BSA-gold.

Aβ1-42 peptides (Sigma) were incubated at 37 °C for 1 h to induce fibril formation in vitro. Grids were inverted onto the drops of Aβ1-42, negatively stained with 2% uranyl acetate, washed with water and dried on filter paper before being viewed by EM.

### Transfection and constructs

Cells were transfected using Lipofectamine 2000 (Invitrogen) for N2a cells or Lipofectamine 3000 (Invitrogen) for primary neurons. N2a cells were transfected in Opti-MEM while primary neurons were transfected directly in their growth medium. The plasmids p3xFLAG-CMV-10-hVPS4A-wt and p3xFLAG-CMV-10-hVPS4A-dn E228Q were generated as described [28]. The control plasmids p3xFLAG-CMV-7-BAP Control Plasmid was purchased from Sigma-Aldrich and pcDNA3-CMV-GFP from Addgene. pcDNA3-synapsin-FLAG-wtVPS4 and pcDNA3-synapsin-FLAG-dnVPS4 were constructed from pcDNA3-synapsin-FLAG and PCR products from the p3xFLAG-CMV-10-hVPS4A-wt and p3xFLAG-CMV-10-hVPS4A-dn respectively. Control plasmid pAAV-synapsin-GFP was purchased from Addgene.

### Antibodies and reagents

The following antibodies were used (see also Additional file 1: Table S1): 369 [29] (Buxbaum et al., 1990) for Western blot (WB) 1:1000; 6E10 (BioLegend, previously Covance SIG-39320) IF: 1:500, WB 1:1000; 12F4 (BioLegend, previously Covance SIG-39142) for immunofluorescence (IF) 1:250; Amyloidβ (1-42) (IBL, 18,582); Amyloidβ (1-42) (Invitrogen, 700,254) IF 1:1000; beta-actin (Sigma, A 5316) WB 1:2000; CD63 (ThermoFisher Scientific, MAI-19281) WB 1:1000; CHMP2B (Abcam, ab33174) IF 1:250, WB 1:1000; Clavestin-1 + 2 (Bioss, bs-6569R-A647) IF 1:250; DAPI (Sigma, D9542) IF 1:2000; drebrin (Abcam, ab11068) IF 1:1000; FLAG (Biolegend, 637,302) IF 1:1000, (Sigma, F1804) WB 1:1000; Flotillin-1 (BD Biosciences, 610,821) IF 1:400; GM130 (BD Biosciences, 610,822) IF: 1:500; GSK3β (Cell Signaling Technology, 12,456) IF 1:400; pGSKα/β (Cell Signaling Technology, 9331S) WB 1:1000; HA-Tag (Cell Signaling Technology, 3724) WB 1:1000; Hrs and Hrs-2 (Enzo, ALX-804-382-C050) IF 1:100; LAMP1 (Abcam, ab24170) IF 1:1000; LAMP1 (Abcam, ab25245) IF: 1:1500; LC3β (Cell Signaling Technology, 2775) WB 1:1000; Amyloid fibrils OC (Merck Millipore, AB2286) IF 1:1000; P2:1 (ThermoFisher Scientific, OMA1-03132) IF 1:500; Phospho-tau pSer396 (ThermoFisher, 44-752G); Rab7 (Abcam, ab50533) IF 1:500; Synaptophysin (Merck Millipore, MAB5258) IF 1:1000; Tsg101 (Genetex, GTX70255) IF 1:250, WB 1:1000; VPS4 (SantaCruz, sc-133,122) IF 1:100, WB 1:1000; secondary antibodies conjugated to Alexa Fluor-488, −546, −647 (IF 1:500; Invitrogen) or to HRP (WB 1:2000; R&D Systems, Minneapolis, MN).

Bafilomycin A (Sigma), torin 1 (Tocris) or rapamycin (Fisher BioReagents) were added to pre-warmed culture media at appropriate concentrations. Starvation media for induction of autophagy was 33% Opti-MEM in Hank’s Balance Salt solution (HBSS). Aβ1-40 or Aβ1-42 peptides (Tocris) were reconstituted in DMSO to 250 μM, sonicated for 10 min and followed by 15 min of centrifugation at 12 k rpm before adding the supernatant to the culture media for the depicted times. All experiments used 0.5 μM of Aβ1-40 or Aβ1-42, except for EM and LAMP-1 positive vesicle size experiments that used 5 μM and 1 μM respectively.

### Cell immunofluorescence

Cultured neurons at 12 DIV or N2a cells were fixed in 4% paraformaldehyde (PFA) in phosphate buffered saline (PBS) with 0.12 M sucrose for 20 min, permeabilized and blocked in PBS containing 2% normal goat serum (NGS), 1% bovine serum albumin (BSA), and 0.1% saponin at room temperature (RT) for 1 h, and then immunolabelled in 2% NGS in PBS overnight at 4 °C. After appropriate washing, coverslips were mounted with SlowfadeGold (Invitrogen). Immunofluorescence was examined by confocal laser scanning microscopy (Leica TCS SP8 or Zeiss LSM 510). In multiple label experiments, channels were imaged sequentially to avoid bleed-through. Images were taken with Leica Confocal Software or Zeiss ZEN software and analysed with ImageJ or Imaris 7.6. LAMP1-positive vesicles in pyramidal neurons were quantified by measuring the diameter of the five largest LAMP1-positive vesicles per cell, imaged by confocal microscopy in z-stacks, (n= >45 LAMP1-positive vesicles). All fluorescent labelling of cells was performed *n* ≥ 3; and in the case of primary neurons from different embryos.

### Brain immunofluorescence

Mice were anesthetized with isoflurane and perfused transcardially with saline followed by 4% PFA in 0.1 M PBS (pH 7.4) at RT. After dissection, brains were postfixed by immersion in 4% PFA in 0.1 M PBS (pH 7.4) at 4 °C for 2 h or overnight. After fixation, brains were cut in 40 μm thick sections with a sliding microtome. Sections were kept in storage buffer composed of 30% sucrose and 30% ethylene glycol in PBS at −20 °C. Free-floating sections were blocked for 1 h in RT with serum and triton-X and then incubated in primary antibodies overnight at 4 °C, followed by appropriate fluorescent Alexa secondary antibodies for 1 h at RT.

### Western blot

Medium was collected and centrifuged and cells were washed twice, harvested in ice cold PBS, and centrifuged. Cell pellets were lysed with 6% sodium dodecyl sulfate (SDS) containing 10 μl/ml β-mercaptoethanol, sonicated, and then heated at 95 °C for 6 min. After centrifugation, supernatants and medium were mixed with loading buffer, heated at 95 °C for 5 min and loaded into 10–20% Tricine gels (Invitrogen). Samples were subjected to electrophoresis and transferred to polyvinylidine difluoride membranes (Millipore). Membranes were blocked in PBS containing 0.1% Tween-20 (PBST) and 5% milk, and incubated in primary antibodies overnight and then with HRP-conjugated secondary antibodies for 1 h diluted in PBS containing 0.1% Tween-20 (PBST) and 5% milk. The immunoreaction was visualized by a chemiluminescence system (Pierce or BioRad). Bands were quantified using Image Lab (Bio-Rad Laboratories). For visualization of Aβ, membranes were boiled in PBS for 5 min prior to blocking. For analysis of exosomes, WB was performed as above but without β-mercaptoethanol in the 6% SDS lysis buffer.

For analysis of LC3β cells were lysed in RIPA buffer (Thermo Fisher Scientific) with protease inhibitor and phosphatase inhibitor (Thermo Fisher Scientific). Lysates with NuPAGE LDS sample buffer and NuPAGE reducing agent were loaded on NuPAGE 4-12% BisTris gels and run with NuPAGE MES SDS buffer (Invitrogen).

For analysis of α-synuclein in medium, total protein was extracted using a trichloroacetic acid (TCA)/acetone precipitation protocol. Briefly, freshly collected samples were cleared by centrifugation at 10000 rpm for 10 min to pellet debris and intact cells. The supernatant was transferred to a new tube and added with ¼ volume of ice-cold 20% TCA followed by incubation on ice for 3 h. The proteins were pelleted by centrifugation at 14000 rpm and washed twice with cold acetone.

For native conditions, cell pellets were lysed on ice in NativePAGE sample buffer (1X, Life Technologies) containing 1% digitonin (Life Technologies) and Halt proteinase inhibitor cocktail (1X, Thermo Scientific) by pipetting up and down and incubating on ice for 15 min. Lysates were centrifuged at 20000 x g for 30 min at 4 °C and protein concentrations of the supernatants were determined with BCA assay. Equal amounts of protein were loaded on a 3-12% NativePAGE Novex Bis-Tris gel (Life Technologies).

### Exosome isolation and analysis

Exosomes were purified from cell culture medium by differential ultracentrifugation as described previously [30]. Briefly, Swe N2a cells were cultured and transfected for 48 h in exosome-free medium. Collected medium was depleted of cells and cellular debris by sequential low speed centrifugation. Exosomes were then isolated by centrifugation of the collected supernatant at 100,000×g at 4 °C for 70 min. The resultant pellet was washed in PBS and centrifuged for 70 min at 100,000×g at 4 °C.

### Statistical analysis

Statistical analysis was performed with PRISM 6 software (Graph-Pad Software, San Diego, CA, USA) by using unpaired t-test or ANOVA with Tukey’s multiple comparisons test or ANOVA with Dunnett’s multiple comparisons test. All data are expressed as the mean ± SD. Differences were considered significant at *p < 0.05, **p < 0.01, ***p < 0.001, ****p < 0.0001.

## Results

### Aβ-dependent MVB enlargement

**Fig. 1:**
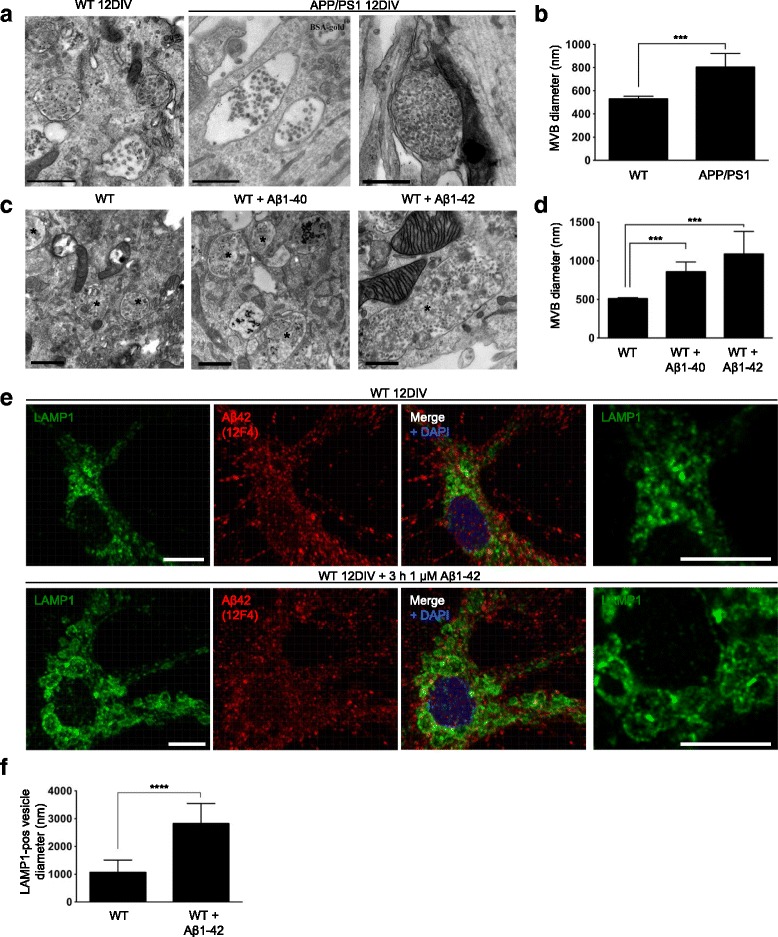
Aβ increases MVB diameter in primary neurons. **a** Electron microscopy reveals that APP/PS1 AD transgenic compared to wt mouse primary neurons have significantly larger MVBs at 12 DIV. Scale bar 500 nm. **b** Quantification of A shows an increase of 52% in MVB diameter in Tg compared to wt neurons, *n* > 94 MVBs per group; *** *p* < 0.001. **c** Exogenously added monomeric Aβ1-40 or Aβ1-42 to wt primary neurons at 12 DIV leads to increased MVB diameter at 48 h on EM images. MVBs are marked with an asterisk. Scale bar 500 nm. **d** Quantification of C, *n* > 45 MVBs per group; *** *p* < 0.001. **e** Shorter time-points of exogenously added monomeric Aβ1-42 already leads to increased size of LAMP1-positive vesicles 3 h after treatment in wt mouse neurons at 12 DIV; 3D-rendering with Imaris from confocal z-stack. High magnification image of LAMP1 labelling from a single focal plane to the right. Scale bar 10 μm. **f** Quantification of E shows significant increase in diameter of LAMP1-positive vesicles after addition of Aβ. The diameters of the five largest LAMP1-positive vesicles per pyramidal neuron were measured in the z-stacks; *n* > 10 neurons per treatment; **** *p* < 0.0001

Endogenous Aβ42 is present in both dendrites and axons of cultured primary APP transgenic neurons and localizes especially with markers of late endosomes/MVBs [21]. In order to examine whether increased levels of Aβ can lead to the enlarged endosomal phenotype seen in AD, MVB size was compared between APP/PS1 transgenic and wt primary mouse neurons. Cells were incubated with BSA-gold 2 h (h) before fixation to confirm identity of the endocytic compartments. Remarkably, electron micrographs showed a significantly greater MVB diameter (52% increase; *p* < 0.001) in APP/PS1 transgenic compared to wt neurons at 12 days in vitro (DIV) (Fig. 1a and b) consistent with the larger diameter of endosomes described in human AD. Since full length APP, β-CTFs, Aβ and the presenilin mutation in the APP/PS1 transgenic neurons potentially all could be the cause of this effect, wt neurons at 12 DIV were treated with freshly prepared human synthetic Aβ1-40 or Aβ1-42 for 48 h, to test if Aβ was sufficient to induce the endosomal enlargements. The neurons demonstrated a 69% and 114% increase in the diameter of MVBs with Aβ1-40 or Aβ1-42 treatment, respectively (*p* < 0.001; Fig. 1c and d) as measured on EM images compared to controls. Further, an increased size by 165% of LAMP1-positive late endosomes/lysosomes was already evident by immunofluorescent labelling at 3 h in pyramidal neurons treated with Aβ1-42 (*p* < 0.0001, Fig. 1e and f). LAMP1-changes at different time points of Aβ1-42 treatment, ranging from 30 s to 48 h, are shown in Additional file 2: Figure S1B. Taken together, our results indicate that enlarged MVBs, one of the earliest pathological features in AD, can be caused by Aβ, which however does not rule out important contributions also of other APP components such as β-CTFs.

### Fibril-like structures in endocytic organelles of wild-type neurons treated with Aβ

**Fig. 2:**
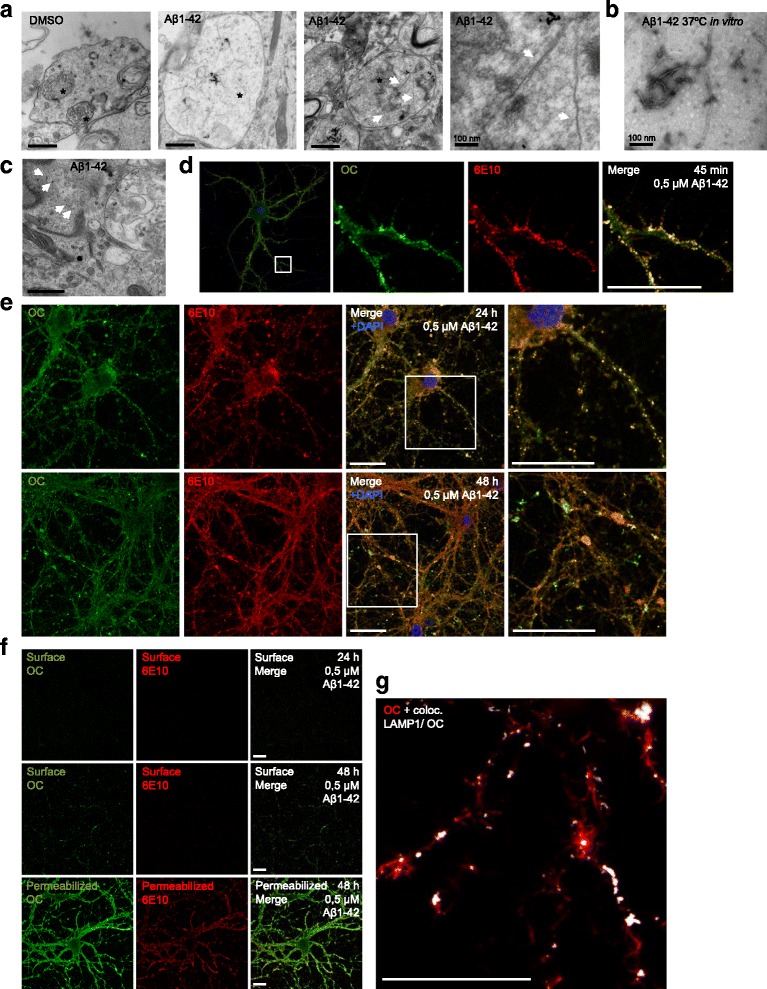
Fibril-like structures in endocytic organelles of wild-type neurons treated with Aβ1-42. **a** Wt primary neurons treated at 12 DIV with Aβ for 48 h induced fibril-like structures in endocytic organelles with Aβ1-42. BSA-gold was added to cells 2 h before fixation to delineate endocytic organelles (*marked with asterisk*). Scale bar 500 nm. High magnification image to the right, scale bar 100 nm. **b** Aβ1-42 peptides incubated in vitro at 37 °C for 1 h to induce fibril formation, imaged with EM. Scale bar 100 nm. **c** BSA-gold (white arrows) was found in the cytosol in Aβ1-42 treated neurons, indicating loss of endolysosomal impermeability due to Aβ1-42. Scale bar 500 nm. **d** OC antibody labelling for fibrillar oligomers and/or fibrils is seen in a vesicular pattern in processes after 45 min treatment with 0.5 μM of Aβ1-42 and colocalizes with human Aβ antibody 6E10 confirming that fibrils consist of the added human Aβ1-42. Scale bar 10 μm. **e** Feeding 0.5 μM of Aβ1-42 to wt primary neurons at 12 DIV at 24 h vs 48 h. At 24 h most of the OC labelling was also antibody 6E10 positive. At 48 h the outer aspects of OC positive structures were 6E10 negative. Scale bar 20 μm. **f** Weak surface labelling of non-permeabilized neurons shows that OC and 6E10 antibody positive structures were intracellular after 24 h of treatment with Aβ1-42 (*upper panel*). At 48 h extracellular OC antibody labelling was now more visible consistent with penetration of the plasma membrane by the elongated OC positive fibrils (*middle panel*). Strong OC and 6E10 antibody labelling of permeabilized cells after fixation but before immunolabelling, shows that the vast majority of added human Aβ1-42 (antibody 6E10) and OC antibody positive fibrils and/or fibrillar oligomers are inside neurons (*lower panel*). Scale bar 20 μm. **g** After 48 h of treatment with 0.5 μM Aβ1-42, OC antibody labelling was seen extending out from LAMP1-positive structures in the processes. The image shows OC with the superimposed colocalizing channel for LAMP1 and OC. For the complete image with separate channels see Additional file 3: S2A. Scale bar 20 μm

Interestingly, fibril-like structures were apparent by EM in endocytic organelles of wt primary neurons incubated with synthetic human Aβ1-42 for 48 h that were not seen in untreated neurons (Fig. 2a), and were more prominent in neurons treated with Aβ1-42 than Aβ1-40. For comparison, Aβ1-42 fibrilized in vitro showed similar size and morphology of fibrils on EM as in the endocytic vesicles (Fig. 2b). Loss of endolysosomal impermeability was also seen with Aβ1-42 treatment of neurons with BSA-gold leaking out into the cytoplasm (Fig. 2c). To further investigate the fibril-like structures, wt murine neurons were treated with elevated levels of human Aβ1-42 at different time points and immunolabelled with the conformational dependent Aβ antibody OC to label amyloid fibrils and fibrillar oligomers [31] and antibody 6E10 to specifically label the added human Aβ. Using the same parameters as in the EM experiments, neurons treated with Aβ1-40 only led to weak OC antibody labelling compared to vehicle treated cells, while Aβ1-42 treated neurons showed robust OC labelling (Additional file 3: Figure S2).

Already at 45 min of treatment with 0.5 μM Aβ1-42 (Fig. 2d) and up to 24 h of incubation, antibodies OC and 6E10 revealed an almost completely overlapping vesicular pattern of labelling, indicating that the amyloid fibrils and/or fibrillar oligomers consisted of Aβ1-42, which was particularly prominent along neuronal processes. Of note, OC labelling increased in intensity with time of treatment with Aβ1-42. Interestingly, at 48 h the OC positive structures were enlarged and elongated, displaying a part that was human Aβ antibody 6E10 positive and an extension that was 6E10 negative (Fig. 2e). Surface labelling of non-permeabilized cells revealed that the elongated OC labelling at 48 h was now to a certain extent extracellular, whereas the punctate OC and 6E10 co-labelling at 24 h was generally intracellular (Fig. 2f). This suggests that with time, fibrils extend out of neurites into the extracellular space and/or that organelles containing the fibrils fuse with the plasma membrane. To better define the subcellular site of Aβ aggregation, cells were double labelled with OC antibody and the late endosomal/lysosomal marker LAMP1. At 45 min colocalization with OC was evident in small LAMP1-positive vesicles in the processes but not in the larger LAMP1-positive vesicles in the cell soma (Additional file 4: Figure S3A). Since lysosomes normally do not localize to axons and dendrites, other than their very proximal part, this suggests that Aβ42 starts to aggregate in late endosomes/MVBs of neurites. At 48 h, OC and LAMP1 still colocalized mainly in neuronal processes, but in LAMP1-positive structures that now appeared somewhat enlarged and irregular in their shape. Elongated OC-positive structures could be seen extending out from the more punctate LAMP1 labelling (Fig. 2g and Additional file 4: Figure S3A). The late endosomal marker Rab7 indicated that at least a subset of the vesicular OC labelling at 24 h colocalized with Rab7 positive late endocytic compartments in neurites (Additional file 4: Figure S3C). Taken together these data indicate that Aβ1-42 can be taken up by neurons in culture and forms amyloidogenic fibrils and/or fibrillar oligomers in late endocytic compartments particularly within neuronal processes that eventually appear to extend extracellularly.

### Aβ1-42 induces changes in the native state of ESCRT-III complex component CHMP2B

**Fig. 3:**
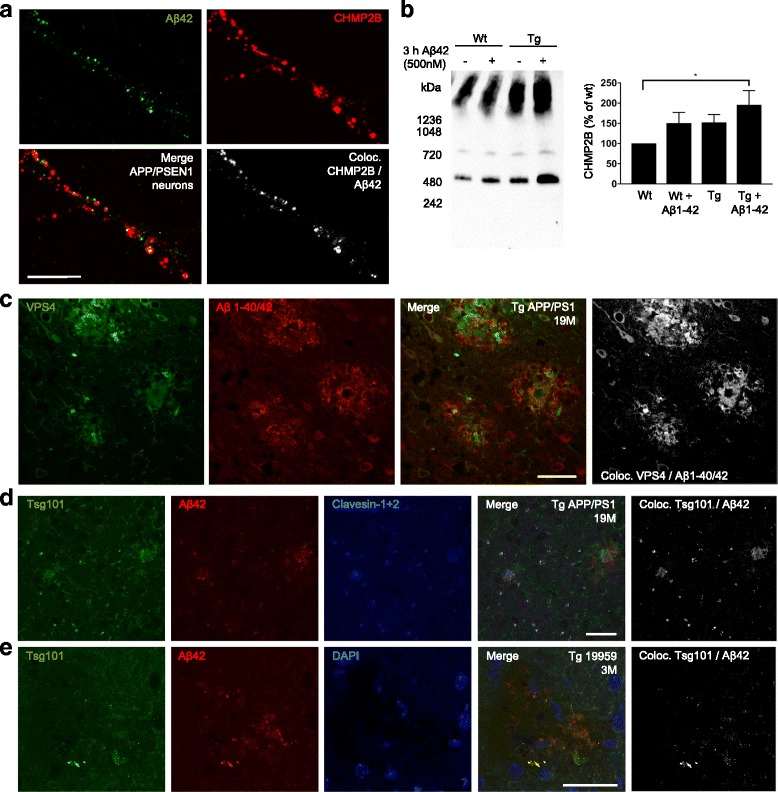
Aβ42 increases high molecular weight complexes of CHMP2B and ESCRT proteins accumulate in amyloid plaques. **a** Immunolabelling of APP/PS1 transgenic primary neurons shows Aβ42 colocalizing with CHMP2B-positive vesicles. Scale bar 10 μm, *n* = 3. **b** A trend for increased high molecular weight complexes of CHMP2B, a 24 kDa ESCRT-III component, in APP/PS1 transgenic compared to wt neurons is seen on native gel. Treatment with 0.5 μM of Aβ1-42 for 3 h further increases these high molecular weight complexes. However only APP/PS1 transgenic neurons treated with Aβ1-42 show a statistically significant increase in high molecular weight complexes compared to wt. Equal amounts of total protein were loaded into the wells, *n* = 6; *p < 0.05. **c** VPS4 colocalizes with Aβ42 in amyloid plaques of 19-month-old APP/PS1 mice. Scale bar 40 μm, *n* = 4. **d** Tsg101 colocalizes with both Aβ42 and the neuronal-specific endo-lysosomal marker clavesin 1 and 2 in amyloid plaques of 19-month-old APP/PS1 mice. Scale bar 40 μm, *n* = 3. **e** In early plaques present in the 3-month-old Tg19959 mice Tsg101 also colocalized with Aβ42. Scale bar 40 μm, *n* = 3

Given prior evidence supporting that sorting via the MVB pathway was impaired by Aβ accumulation in cultured AD transgenic neurons and that Aβ dependent translocation into MVBs seemed affected [21], we next investigated possible dysfunction of the ESCRT pathway in models of AD. In APP/PS1 transgenic primary neurons Aβ42 was found associated with CHMP2B-positive vesicles (Fig. 3a). In young 3-month-old Tg19959 mice, a different transgenic mouse model of β-amyloidosis harbouring the Swedish and Indiana APP mutations, CHMP2B immunolabelling was most prominent in the area of hippocampus and entorhinal cortex that also expressed increased levels of Aβ/APP (Additional file 5: Figure S4A-B). Although levels of total CHMP2B and VPS4 were not significantly changed in wt compared to APP/PS1 primary neurons lysed in 6% SDS (Additional file 5: Figure S4C), levels of high molecular weight complexes of CHMP2B on blue native polyacrylamide gel electrophoresis were increased in APP/PS1 neurons treated with Aβ1-42 for 3 h (Fig. 3b). Other ESCRT proteins were not resolved on native gels, likely due to masking of antibody epitopes under native conditions.

### ESCRT components localize to amyloid plaques

Since AD transgenic neurons showed an increased diameter of MVBs and treatment with Aβ1-42 led to prominent fibril-like structures in late endocytic organelles, and as prior immuno-EM work has indicated early Aβ accumulation in dystrophic neurites [10], we next examined whether aggregating Aβ42 in endosomes might lead to the presence of ESCRT components in amyloid plaques. Indeed, APP/PS1 transgenic mice with plaque pathology demonstrated that the ESCRT-III associated protein VPS4 was markedly increased in plaques (Fig. 3c and Additional file 5: Figure S4D), where it partially colocalized with Aβ42. ESCRT–I component Tsg101, a marker of MVBs, also showed increased labelling in plaques (Fig. 3d). In contrast, labelling of CHMP2B and the earlier ESCRT-0 component Hrs appeared decreased in plaques compared to surrounding brain parenchyma (Additional file 5: Figure S4E-F). Brain sections were also co-labelled with an antibody against clavestin-1 and 2 (Fig. 3d), which are neuron-specific proteins in the endo-lysosomal pathway [32]. The strong colocalization between Tsg101 and clavesin-1 and 2 in amyloid plaques supports the neuronal origin of ESCRT components in plaques. Moreover, much of the strong Aβ42 labelling outside of plaques in AD transgenic brain occurred in vesicle-like structures of dystrophic neurites that also contained clavesin-1 and 2 and Tsg101, consistent with accumulation of Aβ42 within endosomal compartments of neurons. To confirm these results in a different AD transgenic mouse model of β-amyloidosis, Tg19959 mice were examined with the onset of plaque pathology. In early plaques of 3-month-old Tg19959 mice Tsg101 (Fig. 3e) and VPS4 (not shown) also colocalized strongly with Aβ42.

### Blocking VPS4A increases intracellular accumulation and decreases secretion of Aβ

**Fig. 4:**
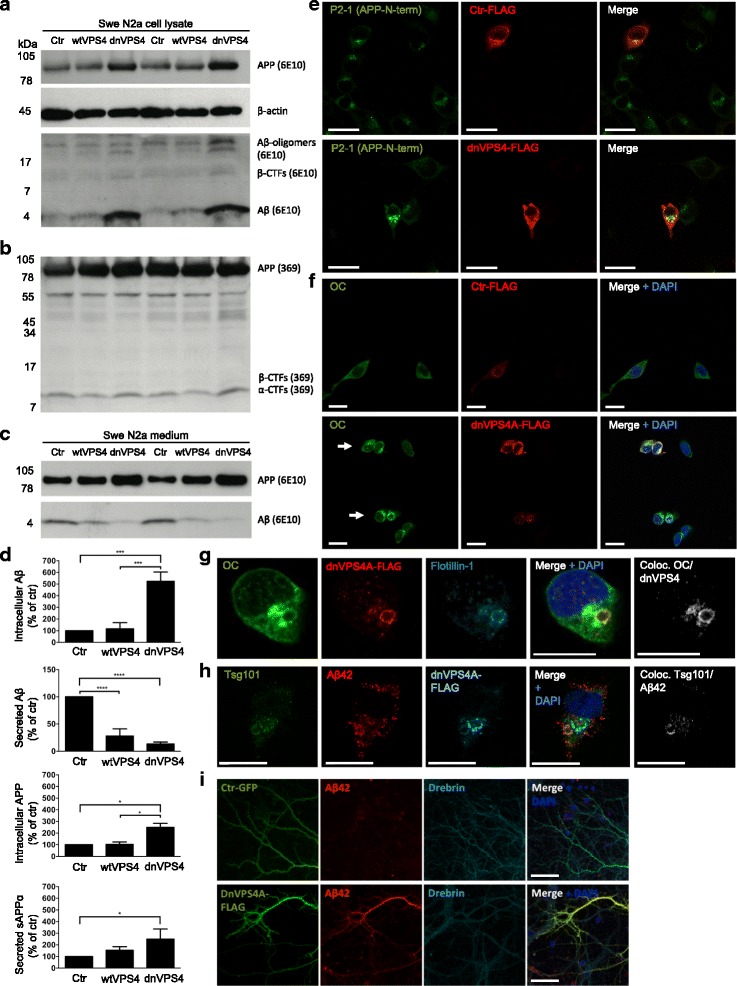
DnVPS4A causes increased accumulation and reduced secretion of Aβ42. Expression of control plasmid (ctr), wtVPS4A (wtVPS4) or the ATP hydrolysis deficient mutant dnVPS4A (dnVPS4) in Swe N2a cells (A-H) or wt primary mouse neurons (I) for 24 h. **a** Representative Western blot of APP, SDS-stable oligomeric Aβ species, β-CTFs and monomeric Aβ in cell lysate probed with antibody 6E10, and β-actin for protein normalization. **b** Western blot analysis of full length APP, β-CTFs and α-CTFs in cell lysate probed with the C-terminal specific APP antibody 369. **c** Western blot analysis of secreted αAPP (sAPPα) and Aβ in cell medium with antibody 6E10. **d** Densitometric quantification of A and C demonstrates that expression of dnVPS4, but not wtVPS4, increases intracellular Aβ compared to ctr. On the other hand, both wtVPS4A and dnVPS4A reduces secreted Aβ compared to ctr, although to a greater extent with dnVPS4A. DnVPS4 increases both cellular and secreted APP. Values are normalized against actin and expressed as percentage of control, *n* > 3; *p < 0.05, **p < 0.01, ***p < 0.001, ****p < 0.0001. **e** Cellular full length APP is increased in cells transfected with dnVPS4A-FLAG (lower panel) compared to control plasmid Ctr-FLAG (*upper panel*). Scale bar 25 μm. **f** Antibody OC labelling of fibrillar oligomers and fibrils is increased in cells transfected with dnVPS4A (*arrows*) compared to control plasmid. Scale bar 25 μm. **g** Antibody OC labelling in dnVPS4-transfected cells is associated with enlarged vesicles positive for flotillin-1. Scale bar 15 μm. **h** Aβ42 accumulation in vesicles positive for ESCRT protein Tsg101 in dnVPS4A-transfected cells. Scale bar 25 μm. **i** Wt primary neurons at 12 DIV transfected with synapsin-dnVPS4A-FLAG or control synapsin-GFP plasmid for 24 h, immunolabelled for Aβ42 and the post-synaptic protein drebrin shows that Aβ42 labelling is increased with expression of dnVPS4A but not with control plasmid. Scale bar 50 μm

In order to model the effect of dysfunctional ESCRT-dependent MVBs on Aβ accumulation, a dominant negative, E228Q ATPase-deficient form of VPS4A (dnVPS4A) was expressed for 24 h in N2a neuroblastoma cells harbouring stably transfected human Swedish mutant APP. N2a cells were used here because of their high transfection efficiency compared to primary neurons. To assess that N2a cells react to Aβ1-42 in a similar manner as primary neurons, N2a cells were treated with exogenously added Aβ1-42 for different time points. N2a cells treated with Aβ1-42 also exhibited increased size of LAMP1-positive vesicles (Additional file 6: Figure S5). The ATPase VPS4 is a key component of the ESCRT machinery as it is the only energy-consuming enzyme, promotes disassembly and recycling of ESCRT-III oligomers, and is recruited to the ESCRT-III complex by direct interaction with CHMP2B. We found that the expression of dnVPS4A markedly increased the intracellular pool of Aβ by 424% (*p* < 0.001, Fig. 4a and d) while concurrently decreasing the amounts of Aβ secreted into the medium by 87% (*p* < 0.0001, Fig. 4c and d). DnVPS4A also increased higher molecular weight bands between 17 kDa and 34 kDa that might represent Aβ oligomers within cells (Fig. 4a) as they were not seen with C-terminal specific APP antibody 369 (Fig. 4b). In addition, dnVPS4A increased the levels of APP within cells by 149% (*p* < 0.05) and secreted APPα in conditioned media by 149% (*p* < 0.05). Overexpression of wild type VPS4A (wtVPS4A) also significantly reduced secretion of Aβ by 72% (*p* < 0.001), although not to the extent of the dominant negative construct. Such a partially dominant negative effect of over-expressing wtVPS4 has been described [28].

Confocal immunofluorescence microscopy of Swe N2a cells transfected with dnVPS4A confirmed the increase of Aβ and full-length APP (Fig. 4e). Increased labelling by immunofluorescent microscopy with the conformational antibody OC in dnVPS4A-transfected cells confirmed the increase in Aβ oligomers and showed that the fibrillar oligomers and/or fibrils labelled by antibody OC colocalized with enlarged vesicles labelled positive for dnVPS4A protein and flotillin-1 (Fig. 4f and g). Triple labelling revealed that Aβ42 accumulated in enlarged vesicles positive for the MVB marker Tsg101 in dnVPS4A expressing cells labelled by the FLAG-tag (Fig. 4h). Thus, dysfunctional MVBs accumulate Aβ42 that, at least in part, is in an oligomeric and/or fibrillar form. In contrast to effects on Aβ and in agreement with a prior report [28], dnVPS4A increased secretion of α-synuclein in α-syn N2a cells without altering the total pool of intracellular α-synuclein (Additional file 7: Figure S6). Since the CMV-FLAG-dnVPS4A induced toxicity in primary neurons, we constructed a plasmid under the weaker synapsin promoter. Expressing dnVPS4A under this synapsin promoter in wt or APP/PS1 primary neurons showed increased labelling of Aβ42 (Fig. 4i), supporting that dysfunctional ESCRT-dependent MVB formation leads to increased levels of Aβ within neurons.

### Increased cellular levels of Aβ with dysfunctional MVBs is mimicked by inhibiting lysosomal degradation

**Fig. 5:**
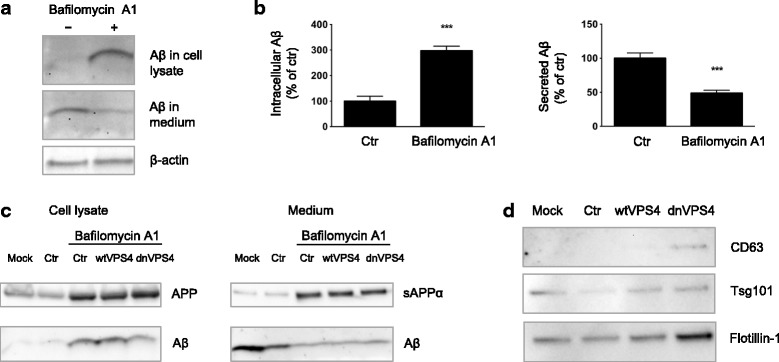
Increased cellular levels of Aβ with dnVPS4A expression is consistent with decreased lysosomal degradation. **a** Bafilomycin A1 (BafA1) treatment had similar effects on Aβ as dnVPS4a, markedly increasing intracellular levels of Aβ and reducing Aβ secretion. Representative Western blot analysis of cell lysates and cell medium from Swe N2a cells treated with 5 nM BafA1 for 24 h and blotted for 6E10 for Aβ and β-actin. **b** Densitometric quantification of A. Values are normalized against actin and expressed as percentage of untreated control, *n* = 3, *** *p* < 0.001. **c** When the ability of lysosomes to degrade Aβ is abolished by BafA1, blocking Aβ on route to the lysosomes via dnVPS4 does not lead to further Aβ accumulation. Representative Western blots are shown of lysates (*left*) and medium (*right*) from Swe N2a cells treated with 5 nM BafA1 6 h after transfection with mock (no plasmid), control plasmid ctrGFP, wtVPS4A or dnVPS4A. Cells were collected 24 h post-transfection, *n* = 3. **d** Western blot analysis of isolated exosomes secreted from Swe N2a cells transfected with mock (no plasmid), ctrGFP, wtVPS4 or dnVPS4 for 48 h. Representative blots demonstrate that exosome secretion is not reduced with dnVPS4. Instead, increased amounts of CD63, an exosomal marker postulated to be involved in ESCRT independent ILV formation, is seen in the exosomal fraction from dnVPS4 transfected cells. Flotillin-1, an exosomal marker associated with cholesterol-enriched lipid rafts is also increased in the exosomal fraction from dnVPS4 transfected cells, while the ESCRT-I protein and exosomal marker Tsg101 is not changed, *n* = 3

The increased intracellular levels of Aβ with dnVPS4A could be due to changes in the production of Aβ, reduced secretion and/or reduced fusion of MVBs with the lysosome for degradation. In order to investigate the turnover of APP and Aβ, Swe N2a were treated with the protein synthesis inhibitor cycloheximide (Additional file 8: Figure S7A-B and Additional file 9: Figure S8D). This revealed a rapid turnover of APP in cell lysates with a half-life of about 45 min, while cellular Aβ had a half-life of about 4 h. In contrast, the degradation of APP and Aβ in conditioned media was slower, not reaching 50% of control levels within 6 h of cycloheximide treatment. To investigate the role of lysosomal degradation, Swe N2a cells were treated for 24 h with 5 nM bafilomycin A1 (BafA1), which inhibits the vacuolar H+ ATPase. BafA1 treatment had the same effects on Aβ as dnVPS4A, markedly increasing intracellular levels of Aβ and reducing the secretion of Aβ (Fig. 5a-b and Additional file 9: Figure S8A-E). These results suggest that dnVPS4A blocks MVBs with Aβ on route to lysosomes for degradation. Consistently, when the endo-lysosomal pH gradient was blocked with BafA1, dnVPS4A transfected cells no longer showed higher cellular levels of Aβ than the wtVPS4A or GFP transfected cells (Fig. 5c). Hence, when the ability of lysosomes to degrade Aβ is abolished, blocking Aβ on route to the lysosomes via dnVPS4 does not lead to further Aβ accumulation.

To investigate the secretion of Aβ via fusion of MVBs with the plasma membrane and concomitant release of exosomes, exosomes from dnVPS4A-transfected Swe N2a cells were isolated and analysed (Fig. 5d). Secretion of exosomes was not prevented but instead increased by dnVPS4A in Swe N2a cells, possibly due to upregulation of ESCRT independent (CD63 dependent) MVB formation [33–35].

### Blocking ESCRT dependent ILV formation increases pathological tau phosphorylation

**Fig. 6:**
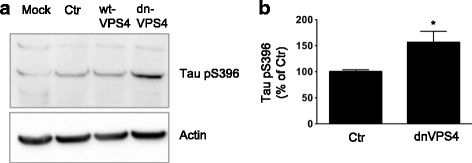
Blocking ESCRT dependent ILV formation increases tau phosphorylation at serine 369 (S396). **a** Western blot of transfected N2a Swe cells demonstrates increased tau phosphorylation at S396 with expression of dnVPS4A. **b** Quantification of A shows a 56% increase in levels tau phosphorylated at S396 with dnVPS4A; values are normalized against actin and expressed as percentage of ctrGFP, (*n* = 5, *p < 0.05)

A major question in AD is how Aβ links to tau pathology. MVBs are necessary for the sequestration of GSK3 [36] which phosphorylates tau at serine 396 (S396). Both elevated tau phosphorylation at S396 [37] and hyperactive GSK3 is implicated in AD [38, 39]. Therefore, we examined the effects of dnVPS4A on tau phosphorylation. Tau phosphorylation at residue S396 was significantly increased by 56% with dnVPS4A (*p* < 0.05; Fig. 6a and b), suggesting increased activation of GSK3β and increased GSK3β dependent phosphorylation of tau caused by dysfunctional ESCRT dependent MVBs. Immunofluorescence microscopy revealed increased GSK3β labelling of cells upon dnVPS4A transfection (Additional file 7: Figure S6A), although changes in total GSK3β or phosphorylated GSK3α/β (S21/9) levels were not detected by Western blot in cell lysates of Swe N2a cells transfected with dnVPS4A (Additional file 7: Figure S6B). One possible explanation for this apparent discrepancy is that the active GSK3β in the cytosol and early endosomes (facing the cytosol) is easier to visualize with immunofluorescence than the sequestered GSK3β inside the ILVs of MVBs.

### Autophagy induction partially rescues the intracellular accumulation of Aβ

**Fig. 7:**
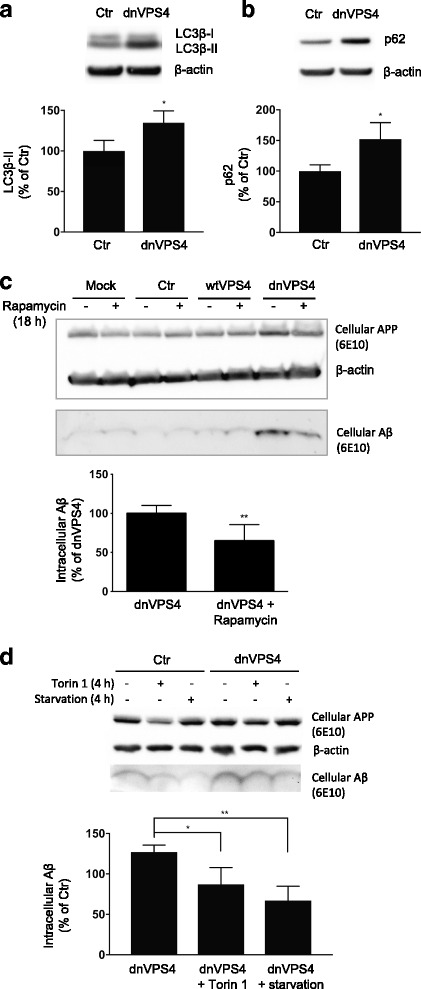
Induction of autophagy partially rescues dnVPS4A induced intracellular accumulation of Aβ. **a** Expression of dnVPS4A increases levels of LC3β-II, an indicator of autophagy, by 35% (*n* = 3, *p < 0.05). Representative blot (above) and quantification (below); values are normalized against actin and expressed as percentage of ctrGFP. **b** dnVPS4A increases levels of p62, an indicator of autophagy, by 52% (*n* = 3, **p < 0.01). Representative blot (above) and quantification (below); values are normalized against actin and expressed as percentage of ctrGFP. **c** Chemically induced autophagy by Rapamycin partially rescues the increase in intracellular Aβ from dnVPS4A. 6 h after transfection Swe N2a cells were treated with 1 μM Rapamycin and harvested 24 h post-transfection. Representative blot (above) and quantification (below) demonstrating 35% reduction in intracellular Aβ by Rapamycin on dnVPS4 transfected cells. Values are normalized against actin and expressed as percentage of dnVPS4 (*n* = 6, **p < 0.01). **d** Induced autophagy by torin 1 or by starvation significantly reduces intracellular Aβ to levels in dnVPS4 transfected cells. Values are normalized against actin and expressed as percentage of control transfected cells (*n* = 3, *p < 0.05, **p < 0.01)

Since autophagic organelles are markedly increased in AD and autophagy is thought to be impaired in the disease [14], we next examined LC3β-II and p62, markers of autophagy, in dnVPS4A transfected cells accumulating intracellular Aβ. Expression of dnVPS4A in Swe N2a cells showed increased levels of LC3β-II, which correlate with increased numbers of autophagosomes in the cell, by 35% (*p* < 0.05, Fig. 7a) and increased levels of p62 by 52% (*p* < 0.05, Fig. 7b). Inducing autophagy by 1 μM Rapamycin (*p* < 0.01, Fig. 7c), 250 nM Torin1 or starvation (Fig. 7d) reduced the dnVPS4A-induced increase in intracellular Aβ. Thus, in the setting of dysfunctional MVBs with Aβ that is inefficiently trafficked to lysosomes for degradation and/or inefficiently secreted, stimulation of autophagy is associated with decreased cellular Aβ.

## Discussion

Over the past years, the view of the role of Aβ in the pathogenesis of AD has been changing. Rather than merely aggregation of extracellular Aβ, a complex and interrelated biology of intra- and extra-cellular pools of Aβ has emerged. Progressive intraneuronal Aβ accumulation and impaired secretion of Aβ were reported in AD transgenic neurons with time in culture [9, 40] and plaque-independent, Aβ-dependent synapse damage and memory impairment correlated with this intracellular pool of Aβ but not plaques in AD-transgenic mice [41]. Our working hypothesis is that dystrophic neurites with accumulating intraneuronal Aβ, initially within MVBs, are a nidus of plaque formation [10], with an important contribution of secreted Aβ originating also from hyperactive neurons.

Here we provide novel molecular insights into endosomal alterations with AD pathogenesis. Enlarged endosomes have been observed to be among the earliest cellular changes in AD and the related AD pathology that develops in Down syndrome [12]. It has been reported that the enlarged phenotype in early endosomes and lysosomes in AD is independent of Aβ and instead only dependent on APP β-CTFs [42, 43]. We now provide evidence that MVB size is increased in AD transgenic neurons, and that this phenotype of increased late endosomal size can be recapitulated in wt neurons with addition of exogenous Aβ. These data support that Aβ can induce endosomal enlargement, but do not exclude an important role also for APP β-CTFs. Although we only measured the size of MVBs with EM, it is likely that also other endocytic compartments including lysosomes and early endosomes were affected. The large LAMP1-positive vesicles seen and measured on confocal images (Fig. 1f) with Aβ treatment, likely also include lysosomes and autolysosomes.

We show by EM fibrillar-like structures inside abnormal MVBs/late endocytic/lysosomal compartments in neurons treated with Aβ1-42, and immunofluorescent labelling further indicates that MVBs/late endocytic compartments contain aggregated Aβ1-42. We cannot fully exclude the possibility that these aggregates begin to form in the cell culture medium and then are taken up by the cells. However, the acidic pH environment and high peptide concentration in a limited space promote amyloid aggregation [44], a milieu that is found inside MVBs. In line with this, it was shown in SHSY5Y cells that synthetic Aβ1-42 added to cell culture medium at 1 μM was taken up and formed aggregates of Aβ inside these cells, while only monomers could be found in the cell culture medium even after 5 days [45]. Overall, these results are consistent with the notion that aggregation of Aβ1-42 is promoted inside acidic endocytic compartments. Friedrich et al. (2010) [46] demonstrated in a macrophage cell line, bundles of Aβ1-40 fibrils in MVBs that penetrated the MVB membrane and leaked into the cytoplasm. We now present the first experimental evidence of Aβ42 aggregates developing inside MVBs/late endocytic/lysosomal compartments of cultured neurons.

We also found evidence of loss of endolysosomal impermeability with Aβ1-42 treatment of neurons, in line with reports in non-neuronal cells [47]. Further, we show that Aβ fibrillar oligomers/fibrils are visible inside neurons in a vesicular pattern as early as 45 min after addition of Aβ1-42 to the cell medium, that are not seen when only labelling the cell surface. Remarkably, at later time points intracellular aggregates are larger and extend into elongated structures that appear to penetrate the plasma membrane or are potentially even secreted or extruded into the extracellular space. Of note, the part of the elongated OC antibody positive structures that developed with time and no longer co-labeled with the human Aβ specific antibody, might represent (1) Aβ where the N-terminal antibody binding sites become inaccessible to the antibody, (2) endogenous mouse Aβ aggregation and/or (3) incorporation of other amyloidogenic proteins. We demonstrate the ESCRT proteins VPS4A and Tsg101 in plaques of two different AD transgenic mouse models. Moreover, these ESCRT proteins strongly colocalized with a neuronal specific marker of the endo-lysosomal pathway, indicating the neuronal origin of the ESCRT proteins in plaques. Previously the lysosomal hydrolases cathepsin D and β-hexosaminidase A were shown to colocalize with Aβ in a subgroup of diffuse plaques of AD and DS patients [48] consistent with an endo-lysosomal origin of aggregated Aβ. However, whether these lysosomal proteins were derived from glial cells or neurons was not determined in that study.

Expression of VPS4 mutants deficient in ATP hydrolysis, such as the dominant negative VPS4 E228Q used in this study, leads to enlarged vesicles defined as a class E phenotype, resulting from disruption of ESCRT-III recycling [49–51]. VPS4 acts after the membrane scission step to recycle ESCRT-III proteins back to monomers, so that they are available to start a second wave of ILV formation [52]. One might speculate that Aβ disturbs this recycling leading to enlarged endocytic vesicles.

We show that inhibition of the late ESCRT machinery component VPS4A mimics AD pathogenesis by causing a marked increase in intracellular accumulation of Aβ and a concomitant decrease in secreted Aβ, consistent with what was reported in cultures of AD-transgenic compared to wt neurons [40]. Choy et al., 2012, reported that depletion of Hrs and Tsg101 in HEK293 cells stably expressing APP695 reduced Aβ secretion [53] and Edgar et al. (2015) found reduced Aβ40 secretion and increased intracellular Aβ when depleting APP overexpressing N2a cells of Hrs or Tsg101 [54], consistent with a role for the ESCRT machinery in preventing intracellular Aβ accumulation. However, in contrast to reduced Aβ secretion on depletion of early ESCRTs, Choy et al. found increased Aβ40 secretion upon VPS4A depletion with siRNA [53]. The difference with our demonstration of reduced Aβ secretion upon expression of dnVPS4 might be explained by the different cell types and methods of altering VPS4A that were used.

We provide evidence that the reduced secretion of Aβ with dnVPS4 was not due to reduced exosome secretion, since total exosome secretion was increased with dnVPS4A. Multiple mechanisms of ILV formation have been identified, but the relationship between different populations of ILVs and MVBs remains unclear. Both ESCRT-dependent and ESCRT-independent mechanisms of MVB biogenesis exist in mammalian cells. A competitive relationship between ESCRT-dependent and -independent mechanisms of ILV formation within single MVBs has been suggested, with upregulation of CD63-dependent ILV formation from ESCRT depletion [33–35]. It was shown in HeLa-CIITA-OVA cells that depletion of VPS4B increased the secretion of CD63 positive exosomes [55], in line with our results of increased amounts of CD63 positive exosomes with dnVPS4A. It is interesting to note that in our EM data from APP/PS1 neurons and in wt neurons treated with Aβ1-42, we saw both enlarged MVBs with many ILVs as well as enlarged MVBs with few ILVs. One can speculate that these might represent two different subsets of MVBs; it is possible that ESCRT-dependent ILV formation is disturbed by Aβ/APP, resulting in enlarged and empty MVBs, and potentially subsequent up-regulation of CD63 dependent ILVs formation resulting in MVBs filled with many ILVs. Others have reported that formation of ILVs destined for exosomal release was not ESCRT dependent, while ESCRTs were necessary for ILVs destined for degradation in the lysosome [56].

The intraneuronal pool of Aβ can have a dual origin, namely the production of Aβ from APP inside neurons and uptake of Aβ from the extracellular space that is secreted by other cells and/or the same neuron. Although we saw a net increase in intracellular Aβ levels with the expression of dnVPS4A supporting impaired degradation of Aβ and APP, we can not rule out that the production of Aβ from APP inside neurons was unaffected. In the OC antibody positive dnVPS4A-transfected cells, the enlarged vesicles also colocalized with increased labelling of flotillin-1 (Fig. 4g), hence associating with cholesterol-enriched lipid microdomains. Interestingly, ATPase-defective mammalian VPS4 was reported to localize to aberrant late endosomes accumulating cholesterol, due to impaired cholesterol trafficking [50] and retention of cholesterol in late endosomal/lysosomal compartments was reported to be associated with alterations in APP processing [57].

Our data also demonstrate that defective MVBs, modelled by dnVPS4A, leads to increased tau phosphorylation at serine 396 (S396). This site is phosphorylated by GSK3β; hence the increased tau phosphorylation could be due to impaired GSK3β sequestration into MVBs. Immunofluorescent labelling of GSK3β was increased with dnVPS4A (Additional file 10: Figure S9A), although total levels of GSK3β or GSK3α/β phosphorylated at serine 21/9 were not changed by Western blot. Hence, we cannot fully conclude that defective sequestration of GSK3β into MVBs is responsible for the increased levels of tau phosphorylation that we see with dnVPS4. However, consistent with our results, Tg APP-V7171 mice with the London mutation were found to have increased phosphorylation of tau at S396 and increased GSK3β activity, but no change in total levels of GSK3β and GSK3β phosphorylated at serine 9 [58].

We show that Aβ aggregation can initiate inside nerve cells from vesicular accumulation of Aβ. Aberrant endosomal trafficking has been linked genetically and biologically to a number of neurodegenerative diseases. Proteins involved in endocytosis are also prominent among genes linked to AD [11]. Interestingly, CD2AP, which is genetically linked to late onset AD and has been reported to affect MVB biogenesis and ILV formation [59], was recently reported to elevate levels of intracellular Aβ in dendrites [60]. While the ESCRT-III protein CHMP2B was first genetically linked to FTD [23], copy number variation in CHMP2B has since been reported in a family with familial Alzheimer’s disease [24] and genome-wide association studies for late onset AD identified an association with VPS4B [25]. Moreover, immunoreactivity for CHMP2B is increased in neurons of hippocampus in another characteristic neuropathology of AD, granulovacuolar degeneration (GVD) [61]. CHMP2B-positive GVDs were reported to colocalize to a greater extent with the late endosomal/lysosomal marker LAMP1 than to the lysosomal marker cathepsin D or to the autophagic markers LC3 and p62, suggesting a late endosomal origin of GVDs or that they accumulate at the nexus of autophagic and endocytic pathways [62]. It is interesting to note that we found CHMP2B immunoreactivity particularly in hippocampus and medial temporal lobe of 3-month-old Tg19959 mice before plaque pathology, the two areas that are the first to have GVD-affected neurons in AD [63].

## Conclusions

Neuropathological studies have pointed to an early and aberrant accumulation and aggregation of Aβ within neurons in AD, in particular in dystrophic neurites. Cell biological studies that model this aggregation in neurons are valuable in delineating the molecular mechanisms of Aβ-related synaptic dysfunction. We propose a model where elevated levels of Aβ42 cause enlarged and defective MVBs, possibly via effects on ESCRT-III components. Alternatively, MVB dysfunction, as modelled by the expression of dnVPS4A, can lead to accumulation of Aβ in enlarged endocytic compartments. These results support a scenario where disturbances in the MVB pathway caused by Aβ42, or vice versa, could turn into a vicious cycle where more Aβ42 accumulates and oligomeric and fibrillar aggregates form. Our findings that ESCRT components colocalize with Aβ42 in amyloid plaques in two different mouse models of AD support the scenario that aggregated Aβ42 in MVBs/late endocytic compartments, potentially together with ESCRT-components could serve as seeds for plaques.

## Additional files

Additional file 1: Table S1. List of antibodies. (PDF 5545 kb)

Additional file 2: Figure S1. (A) EM image of an enlarged MVB in wt neurons treated with Aβ1-42. This image shows an example of an enlarged MVB with very few ILVs. (B) Confocal analysis of changes in size of LAMP1-positive structures in Aβ1-42 treated wt primary neurons with time. Scale bar 40 μm. (PDF 34 kb)

Additional file 3: Figure S2. (A) Confocal analysis of wt primary neurons show that untreated (DMSO) cells have no OC labelling, while cells incubated with Aβ1-40 for 48 h have low levels of OC labelling. However, cells incubated with Aβ1-42 display very strong OC labelling. (PDF 8957 kb)

Additional file 4: Figure S3. (A) The early relatively weak OC labelling at 45 min of Aβ1-42 treatment colocalizes with LAMP1 labelling in the neurites, but not with the large LAMP1-positive structures in the cell soma. Confocal analysis of wt primary neurons treated with Aβ1-42 for 45 min. Scale 20 μm. (B) After 48 h of Aβ1-42 treatment, OC labelling is stronger and colocalizes partly with LAMP1-positive structures that appear enlarged and irregular in their shape. Elongated OC-positive structures extend out from such punctate LAMP1 labelling in the neuronal processes. Scale 20 μm. (C) At high magnification, antibody OC labelling can be seen colocalizing with the late endocytic marker Rab7 in neuronal processes of wt neurons treated for 24 h with Aβ1-42. Scale bar 5 μm. (PDF 4688 kb)

Additional file 5: Figure S4. ESCRT proteins in primary neurons and plaques. (A-B) In young 3-month-old Tg19959 mice, CHMP2B immunolabelling is increased in areas of hippocampus (a) and entorhinal cortex (b) that also have increased labelling of APP/Aβ (6E10). Scale bar 40 μm, *n* = 4. (C) Western blot analysis of APP/PS1 compared to wt primary neurons at 12 DIV lysed in 6% SDS shows that protein levels of CHMP2B and VPS4 are not significantly changed, although there is a trend for increased levels of CHMP2B in APP/PS1 neurons, *n* > 6. Protein levels are expressed as percentage of control and are corrected against actin. (D) VPS4 colocalizes with Aβ42 in a vesicular pattern in 19-month-old wt mice (upper panel), *n* = 3. In 19-month-old APP/PS1 mice VPS4 accumulates in and around amyloid plaques (lower panel, white arrows). Scale bar 40 μm, *n* = 4. (E) Decreased labelling of CHMP2B in plaques (white arrows) in 19-month-old APP/PS1 mice. Some colocalization of CHMP2B is seen in Aβ/APP (6E10) positive cells (grey arrows). Scale bar 40 μm, *n* = 3. (F) Labelling of early ESCRT-0 component Hrs is decreased in amyloid plaques compared to surrounding brain parenchyma. Scale bar 40 μm, *n* = 2. (PDF 269 kb)

Additional file 6: Figure S5. Aβ1-42 increases the diameter of LAMP-1 positive vesicles in N2a cells. Confocal images of exogenously added monomeric Aβ1-42 incubated for different time points, ranging from 15 min to 48 h, in N2a cells. 3D-rendering with Imaris from confocal z-stack. Colocalization of OC labelling and LAMP1 labelling can be seen from 45 min of Aβ treatment. The last image is from a single focal plane showing OC labelling inside an enlarged LAMP1-positive structure as well as OC labelling that appears to localize at the cell surface. Scale bar 10 μm. (PDF 13678 kb)

Additional file 7: Figure S6. DnVPS4A increases secretion but does not change levels of intracellular α-syn. (A) Western blot analysis of α-syn N2a cells transfected with dnVPS4A shows increased levels of extracellular α-synuclein without altering the total pool of intracellular α-synuclein. (B) Quantification of A. Values are normalized against actin and expressed as percentage of control, *n* = 3; *p < 0.05, **p < 0.01. (C) Overexposed WB membrane for secreted α-synuclein (same as above) with increased intensity to better visualize the bands. (PDF 375 kb)

Additional file 8: Figure S7. (A) Western blot analysis of APP and Aβ in Swe N2a cells treated with 40 μg/ml cycloheximide (CHX) at different times in hours (h) before harvest. Cell culture media was replaced with fresh media 24 h before harvest. For quantification, values are normalized against actin and expressed as percentage of control, *n* = 3; *p < 0.05, **p < 0.01, ***p < 0.001, ****p < 0.0001 (ANOVA with Dunnett’s multiple comparisons test, compared to ctr). (B) Confocal images of 6E10 and Golgi marker GM130 in Swe N2a cells treated with 40 μg/ml CHX for the depicted times. (PDF 1580 kb)

Additional file 9: Figure S8. (A) Western blot analysis of APP and Aβ in Swe N2a cells treated with 5 nM bafilomycin A1 (BafA1) at different time points (h) before harvest. Cell culture media was replaced with fresh media 24 h before harvest. (B) Quantification of A. Values are normalized against actin and expressed as percentage of control, *n* = 3; *p < 0.05, **p < 0.01, ***p < 0.001, ****p < 0.0001 (ANOVA with Dunnett’s multiple comparisons test, compared to ctr). (C) Confocal images of 6E10 and LAMP1 labelling in Swe N2a cells treated with 5 nM bafilomycin A1 for the depicted times. At 24 h there is a build up of both 6E10 labelling and punctate LAMP1-positive structures. (D) Western blot analysis of APP and Aβ in Swe N2a cells treated with 5 nM bafilomycin A1 (BafA1) 24 h before harvest and 40 μg/ml cycloheximide (CHX) at different time points (h) before harvest. Cell culture media was replaced with fresh media 24 h before harvest, before the addition of Baf A1. (PDF 2264 kb)

Additional file 10: Figure S9. (A) 3D images show increased GSK3β labelling in dnVPS4-expressing N2a Swe compared to cells transfected with control plasmid. Rab7 labelling is also increased in dnVPS4 expressing cells. Scale bar 15 μm. (B) Western blot analysis of cell lysates of Swe N2a cells transfected with dnVPS4A showing no changes in total GSK3β or phosphorylated GSK3α/β (serine 21/9). (PDF 1315 kb)

## Abbreviations

AD: Alzheimer’s disease
APP: Amyloid precursor protein
APP/PS1: B6.Cg-Tg (APPswe,PSEN1dE9)85Dbo/Mmjax mice
BACE1: Beta-secretase 1
BafA1: Bafilomycin A1
BSA: Bovine serum albumin
DIV: Days in vitro
dnVPS4A: Dominant negative VPS4A
EM: Electron microscopy
ESCRT: Endosomal sorting complexes required for transport
FTD: Frontotemporal dementia
ILV: Intraluminal vesicle
MVB: Multivesicular bodies
NFTs: Neurofibrillary tangles
NGS: Normal goat serum
PBS: Phosphate buffered saline
PBST: PBS containing 0.1% Tween-20
PFA: Paraformaldehyde
PS1: Presenilin 1
RT: Room temperature
SDS: Sodium dodecyl sulfate
TCA: Trichloroacetic acid
tg: AD transgenic
wtVPS4A: Wild type VPS4A
