# Supplementary figures and images for: Aβ accumulation causes MVB enlargement and is modelled by dominant negative VPS4A
Source: Mol Neurodegener. 2017 Aug 23;12:61. doi: 10.1186/s13024-017-0203-y (PMC5569475; doi:10.1186/s13024-017-0203-y)

Figure S8

A

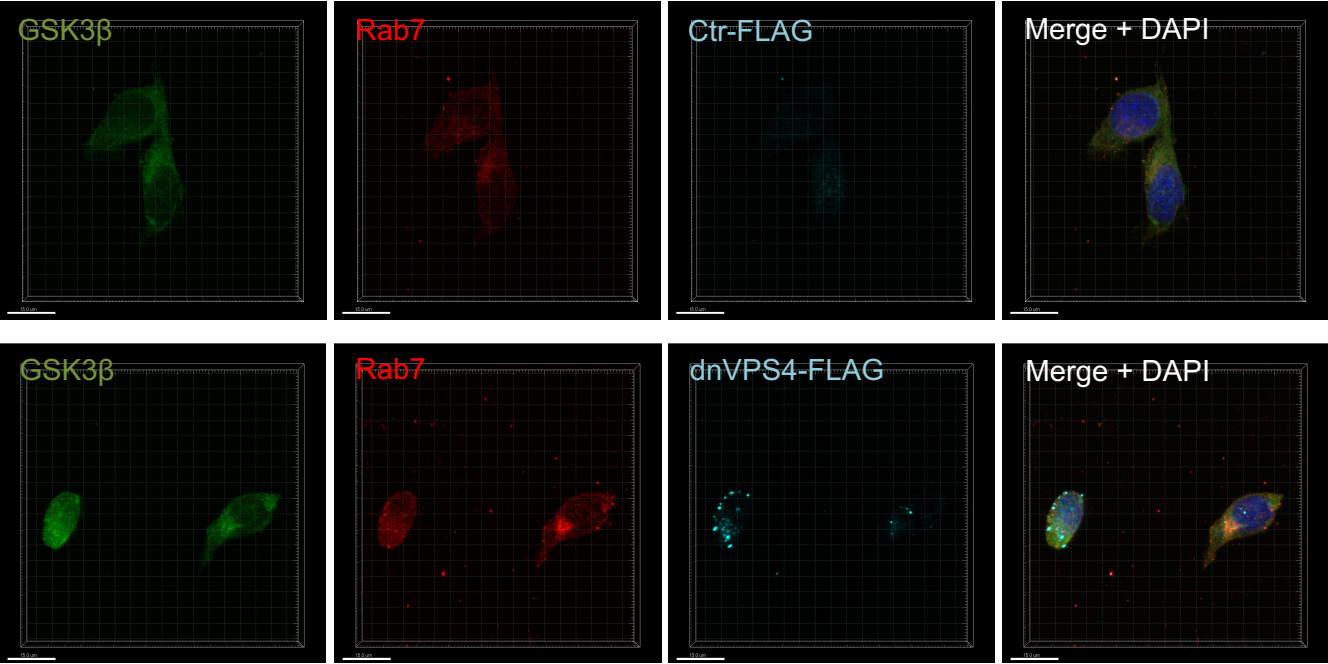

B

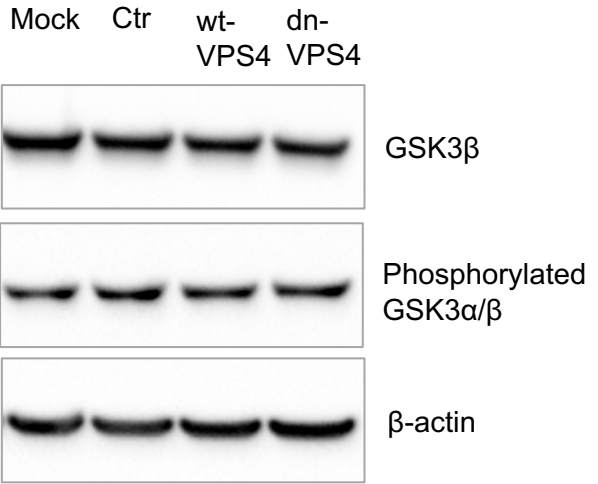

Supplement: Supplementary file 1 — List of antibodies. (PDF 5545 kb) [file 13024_2017_203_MOESM10_ESM.pdf]

Figure S1

WT + A $\beta$ 1-42

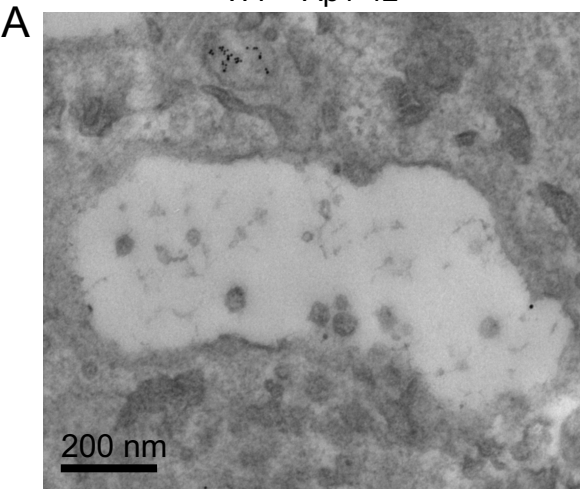

B

LAMP1 + DAPI

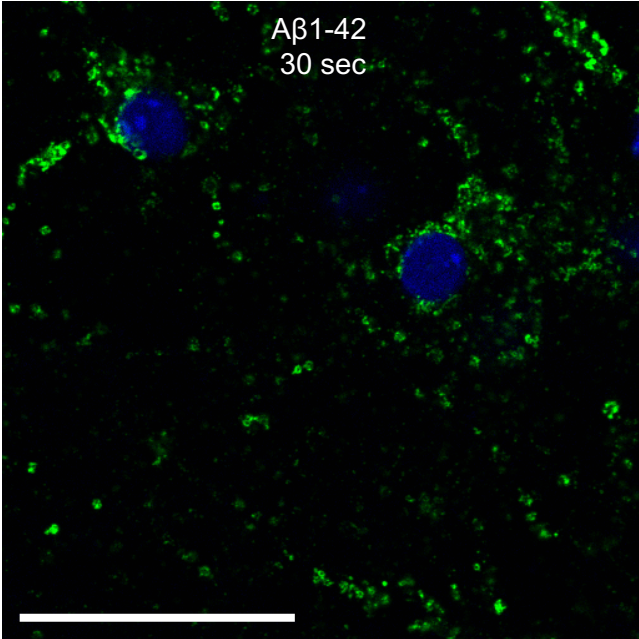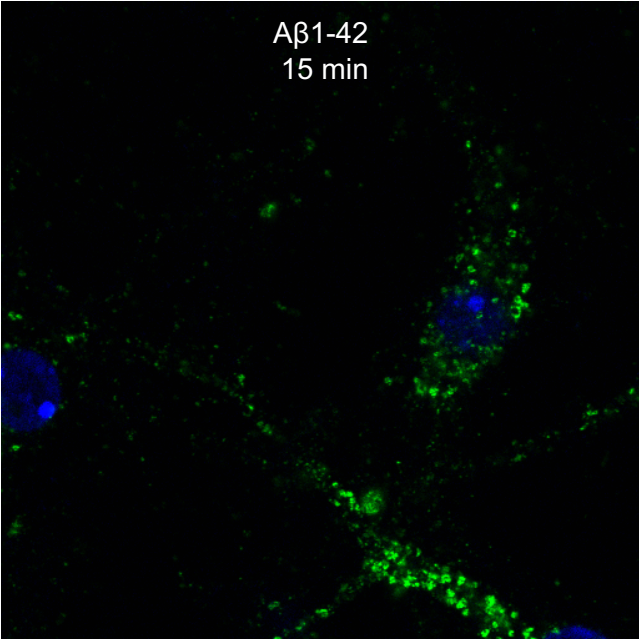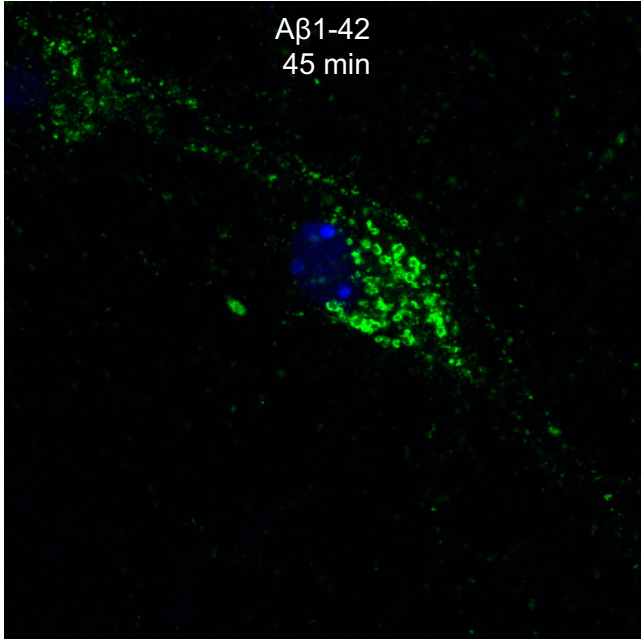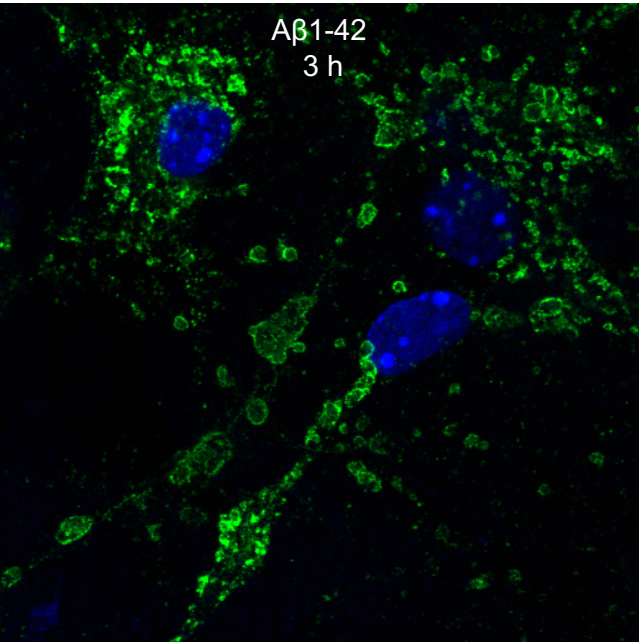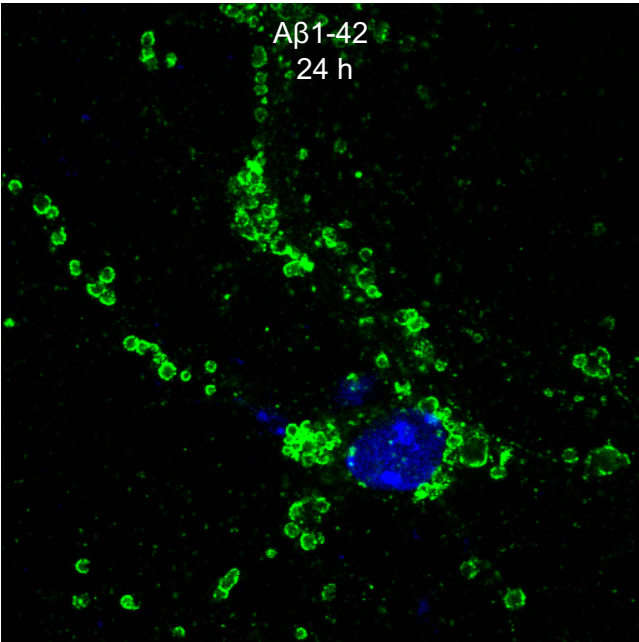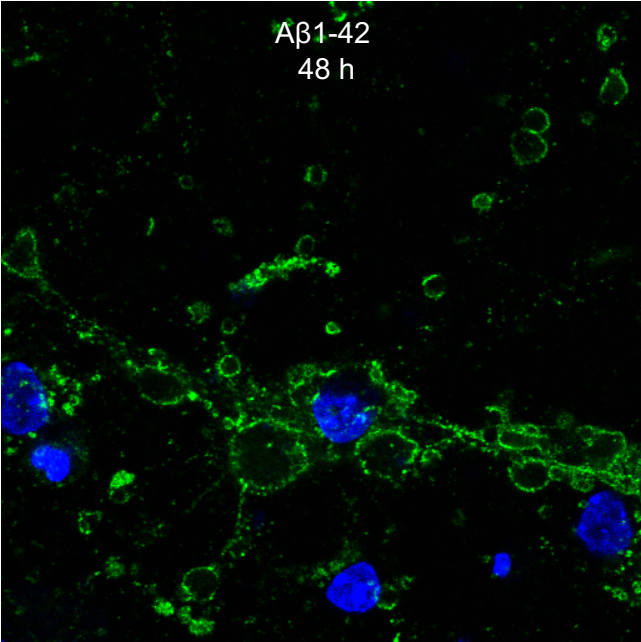

Supplement: Supplementary file 3 — (A) Confocal analysis of wt primary neurons show that untreated (DMSO) cells have no OC labelling, while cells incubated with Aβ1-40 for 48 h have low levels of OC labelling. However, cells incubated with Aβ1-42 display very strong OC labelling. (PDF 8957 kb) [file 13024_2017_203_MOESM2_ESM.pdf]

Figure S2

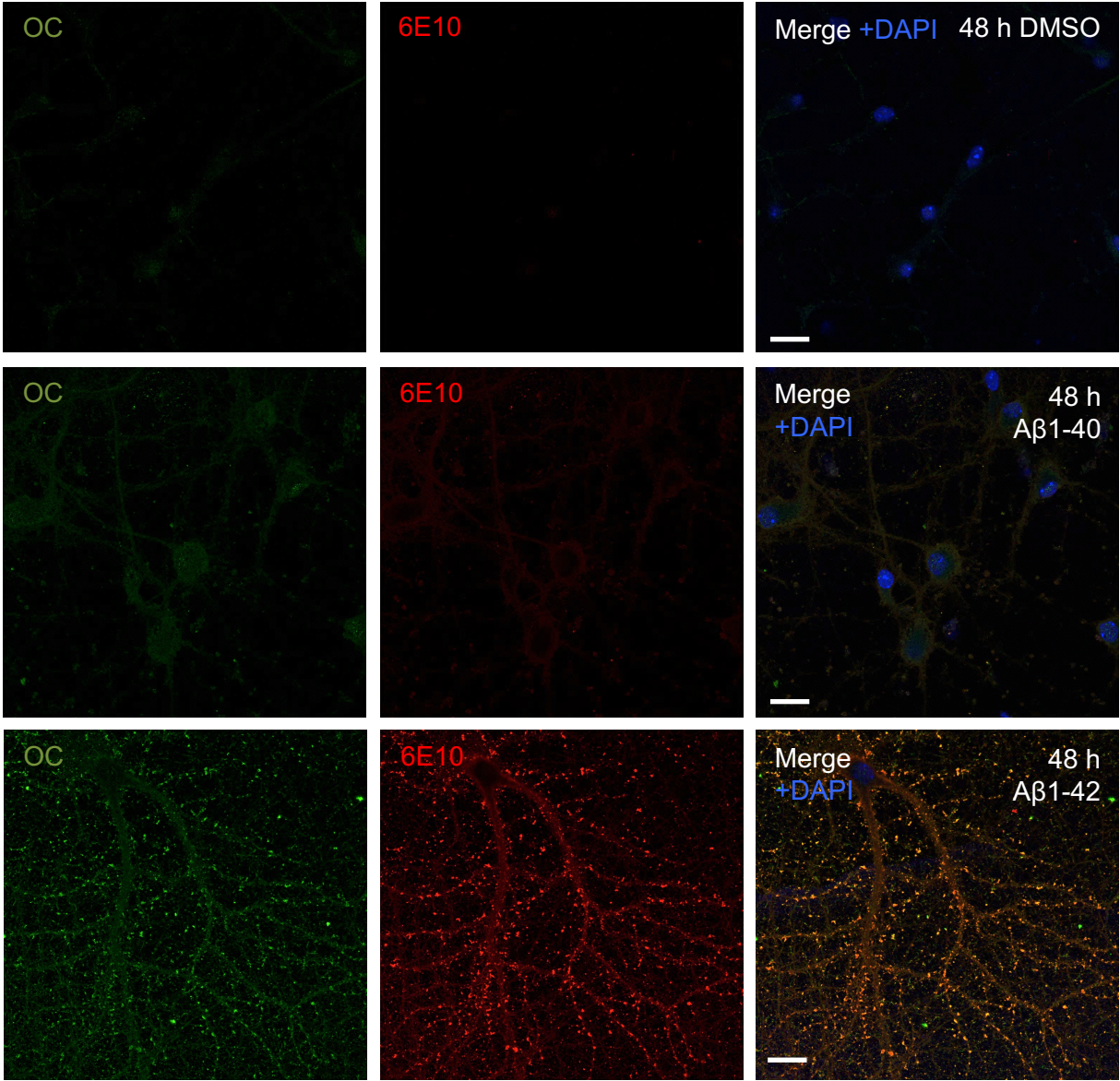

Supplement: Supplementary file 4 — (A) The early relatively weak OC labelling at 45 min of Aβ1-42 treatment colocalizes with LAMP1 labelling in the neurites, but not with the large LAMP1-positive structures in the cell soma. Confocal analysis of wt primary neurons treated with Aβ1-42 for 45 min. Scale 20 μm. (B) After 48 h of Aβ1-42 treatment, OC labelling is stronger and colocalizes partly with LAMP1-positive structures that appear enlarged and irregular in their shape. Elongated OC-positive structures extend out from such punctate LAMP1 labelling in the neuronal processes. Scale 20 μm. (C) At high magnification, antibody OC labelling can be seen colocalizing with the late endocytic marker Rab7 in neuronal processes of wt neurons treated for 24 h with Aβ1-42. Scale bar 5 μm. (PDF 4688 kb) [file 13024_2017_203_MOESM3_ESM.pdf]

Figure S3

A $\beta$ 1-42 45 min

A

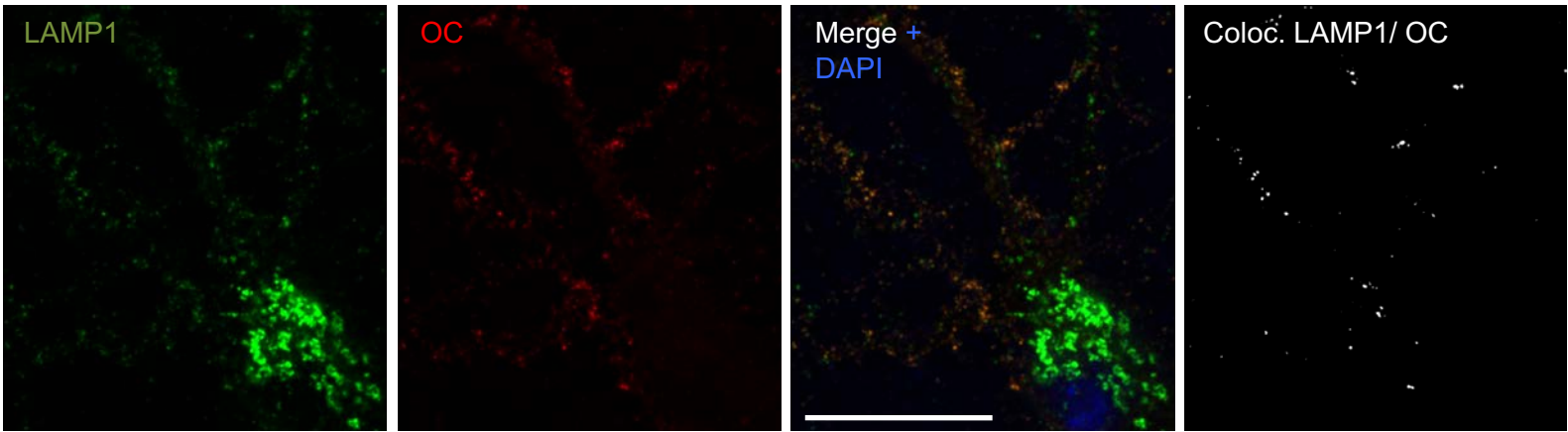

A $\beta$ 1-42 48 h

B

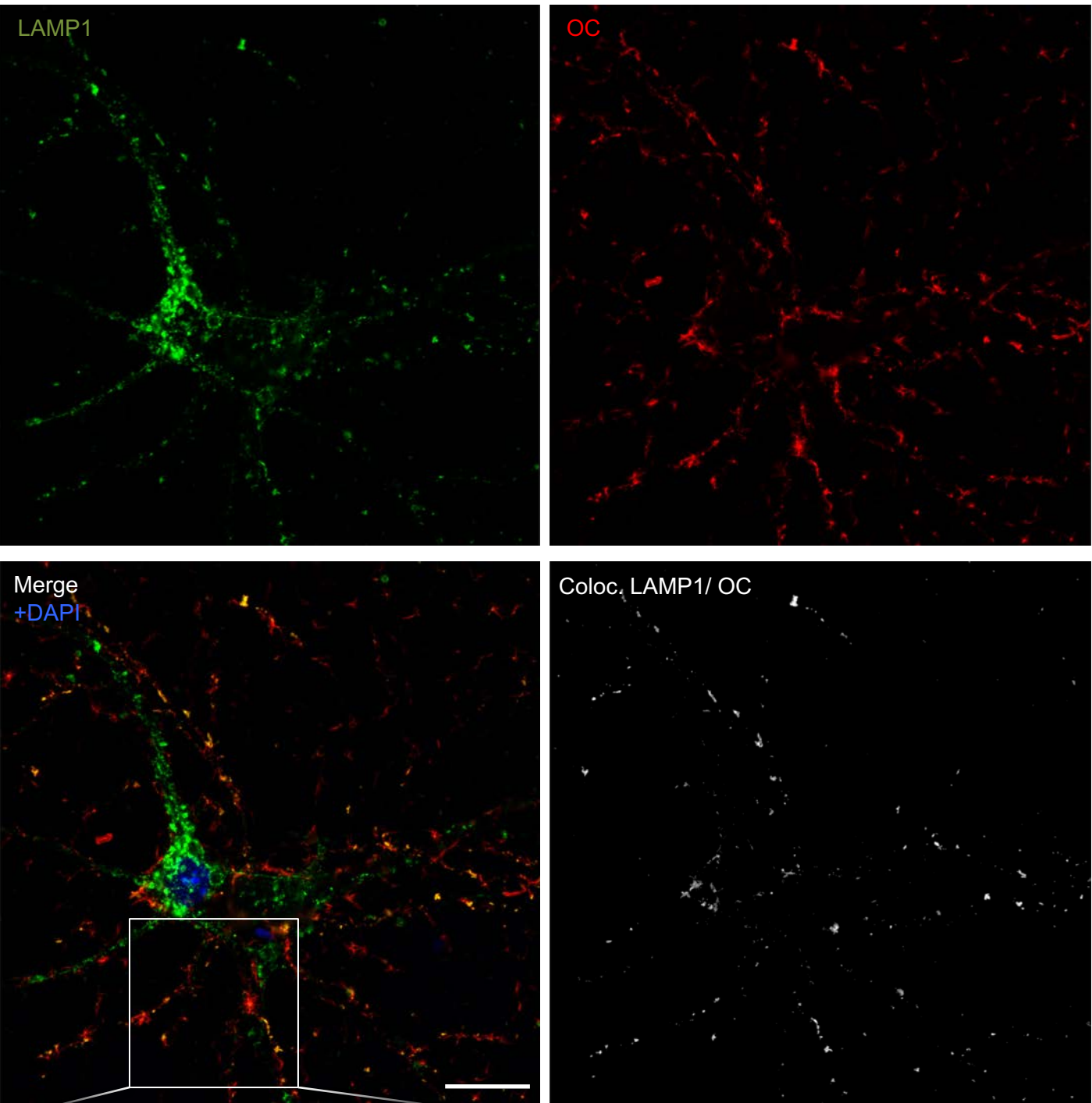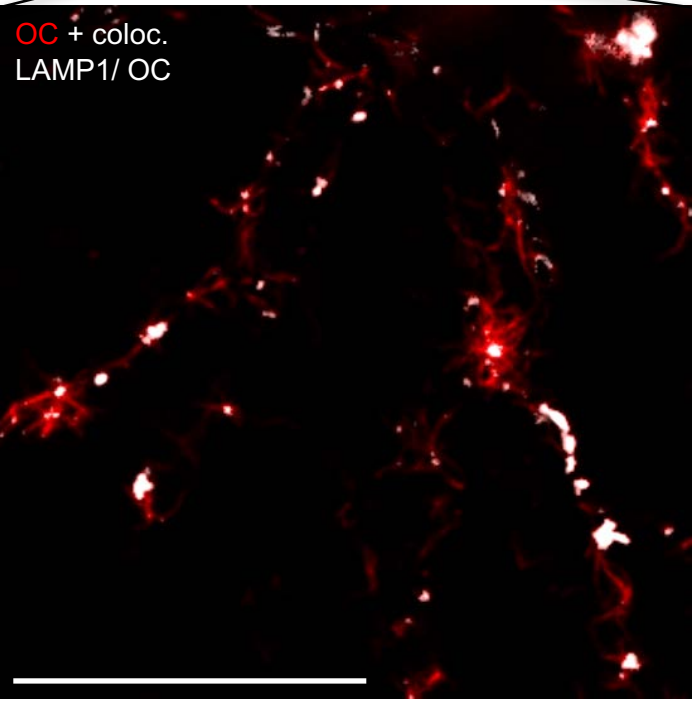

C

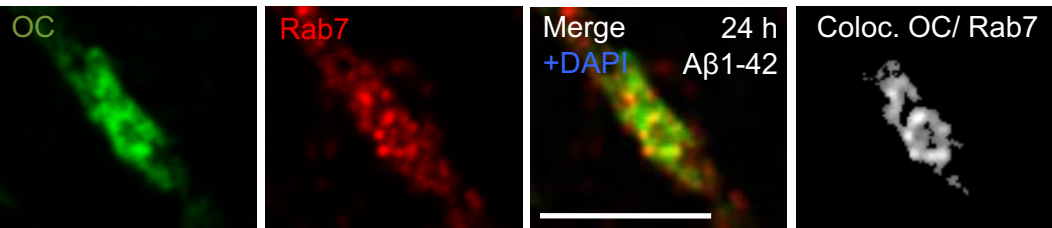

Supplement: Supplementary file 5 — ESCRT proteins in primary neurons and plaques. (A-B) In young 3-month-old Tg19959 mice, CHMP2B immunolabelling is increased in areas of hippocampus (a) and entorhinal cortex (b) that also have increased labelling of APP/Aβ (6E10). Scale bar 40 μm, n = 4. (C) Western blot analysis of APP/PS1 compared to wt primary neurons at 12 DIV lysed in 6% SDS shows that protein levels of CHMP2B and VPS4 are not significantly changed, although there is a trend for increased levels of CHMP2B in APP/PS1 neurons, n > 6. Protein levels are expressed as percentage of control and are corrected against actin. (D) VPS4 colocalizes with Aβ42 in a vesicular pattern in 19-month-old wt mice (upper panel), n = 3. In 19-month-old APP/PS1 mice VPS4 accumulates in and around amyloid plaques (lower panel, white arrows). Scale bar 40 μm, n = 4. (E) Decreased labelling of CHMP2B in plaques (white arrows) in 19-month-old APP/PS1 mice. Some colocalization of CHMP2B is seen in Aβ/APP (6E10) positive cells (grey arrows). Scale bar 40 μm, n = 3. (F) Labelling of early ESCRT-0 component Hrs is decreased in amyloid plaques compared to surrounding brain parenchyma. Scale bar 40 μm, n = 2. (PDF 269 kb) [file 13024_2017_203_MOESM4_ESM.pdf]

Figure S4

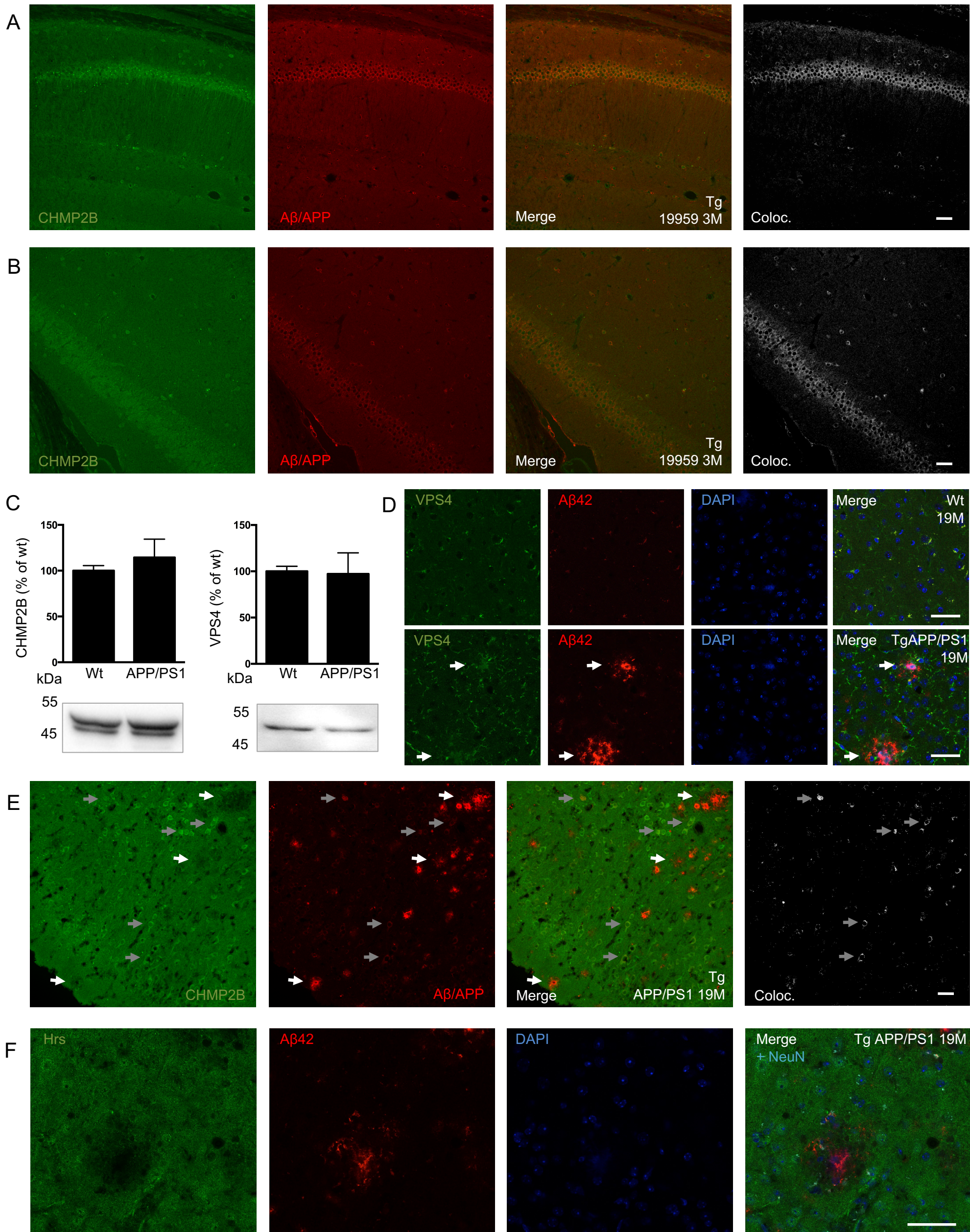

Supplement: Supplementary file 6 — Aβ1-42 increases the diameter of LAMP-1 positive vesicles in N2a cells. Confocal images of exogenously added monomeric Aβ1-42 incubated for different time points, ranging from 15 min to 48 h, in N2a cells. 3D-rendering with Imaris from confocal z-stack. Colocalization of OC labelling and LAMP1 labelling can be seen from 45 min of Aβ treatment. The last image is from a single focal plane showing OC labelling inside an enlarged LAMP1-positive structure as well as OC labelling that appears to localize at the cell surface. Scale bar 10 μm. (PDF 13678 kb) [file 13024_2017_203_MOESM5_ESM.pdf]

Figure S5

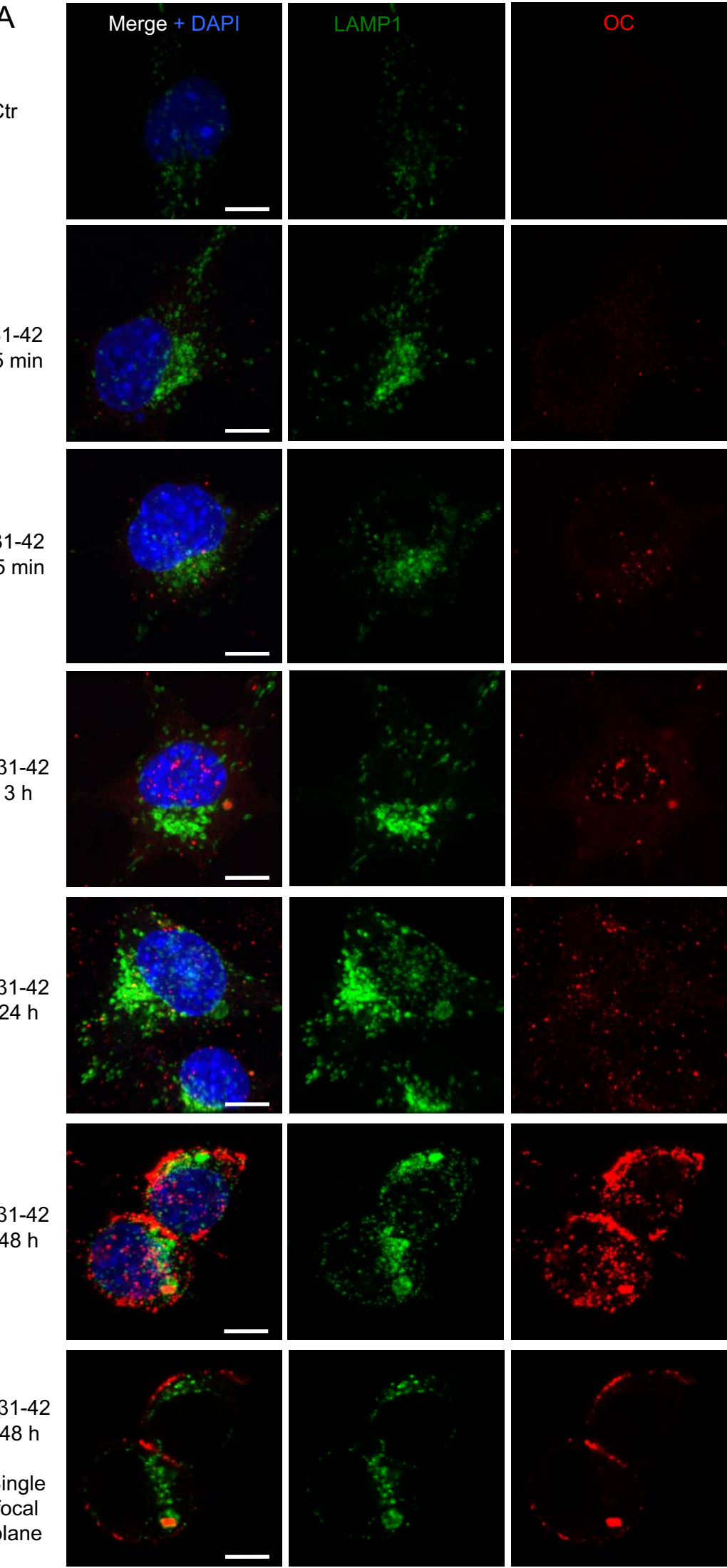

Supplement: Supplementary file 7 — DnVPS4A increases secretion but does not change levels of intracellular α-syn. (A) Western blot analysis of α-syn N2a cells transfected with dnVPS4A shows increased levels of extracellular α-synuclein without altering the total pool of intracellular α-synuclein. (B) Quantification of A. Values are normalized against actin and expressed as percentage of control, n = 3; *p < 0.05, **p < 0.01. (C) Overexposed WB membrane for secreted α-synuclein (same as above) with increased intensity to better visualize the bands. (PDF 375 kb) [file 13024_2017_203_MOESM6_ESM.pdf]

Figure S5

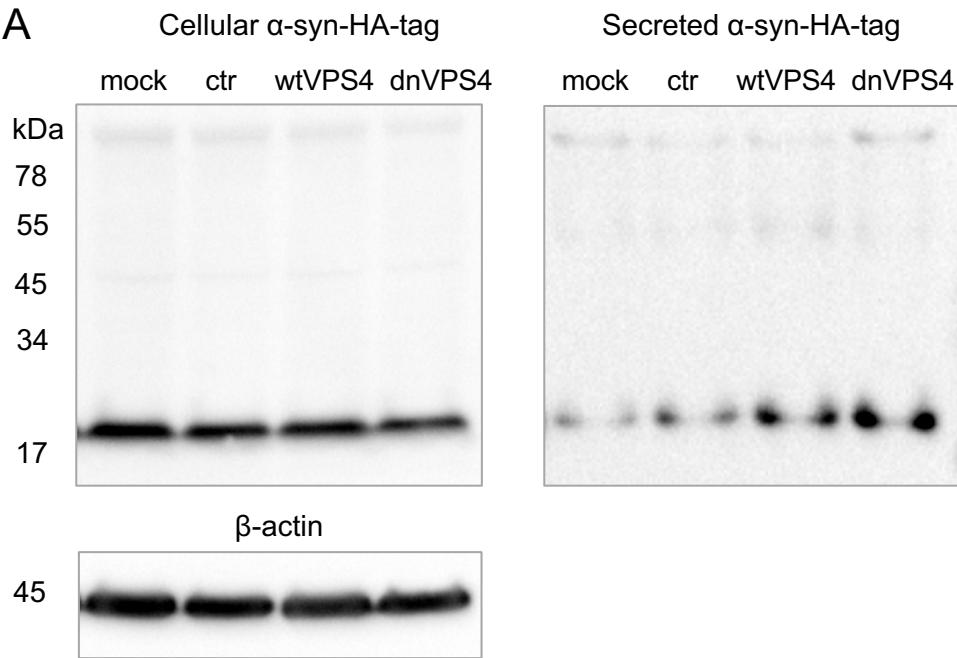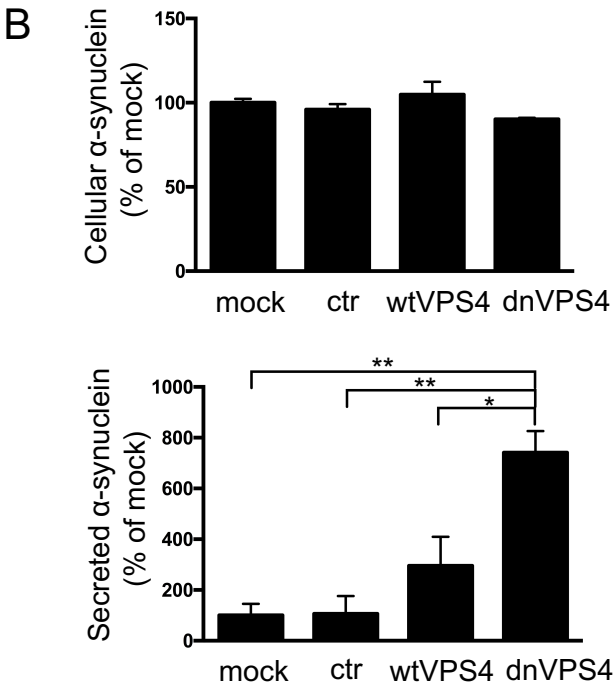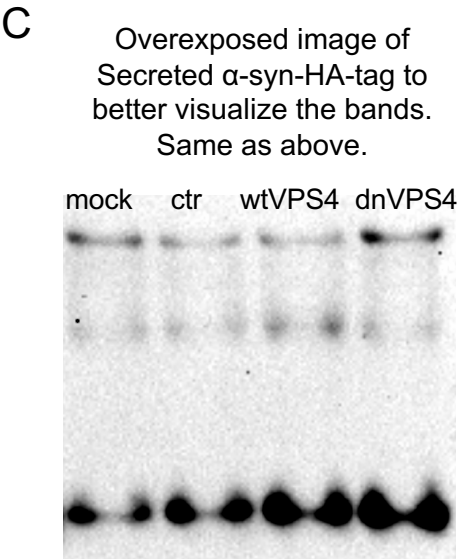

Supplement: Supplementary file 8 — (A) Western blot analysis of APP and Aβ in Swe N2a cells treated with 40 μg/ml cycloheximide (CHX) at different times in hours (h) before harvest. Cell culture media was replaced with fresh media 24 h before harvest. For quantification, values are normalized against actin and expressed as percentage of control, n = 3; *p < 0.05, **p < 0.01, ***p < 0.001, ****p < 0.0001 (ANOVA with Dunnett’s multiple comparisons test, compared to ctr). (B) Confocal images of 6E10 and Golgi marker GM130 in Swe N2a cells treated with 40 μg/ml CHX for the depicted times. (PDF 1580 kb) [file 13024_2017_203_MOESM7_ESM.pdf]

Figure S7

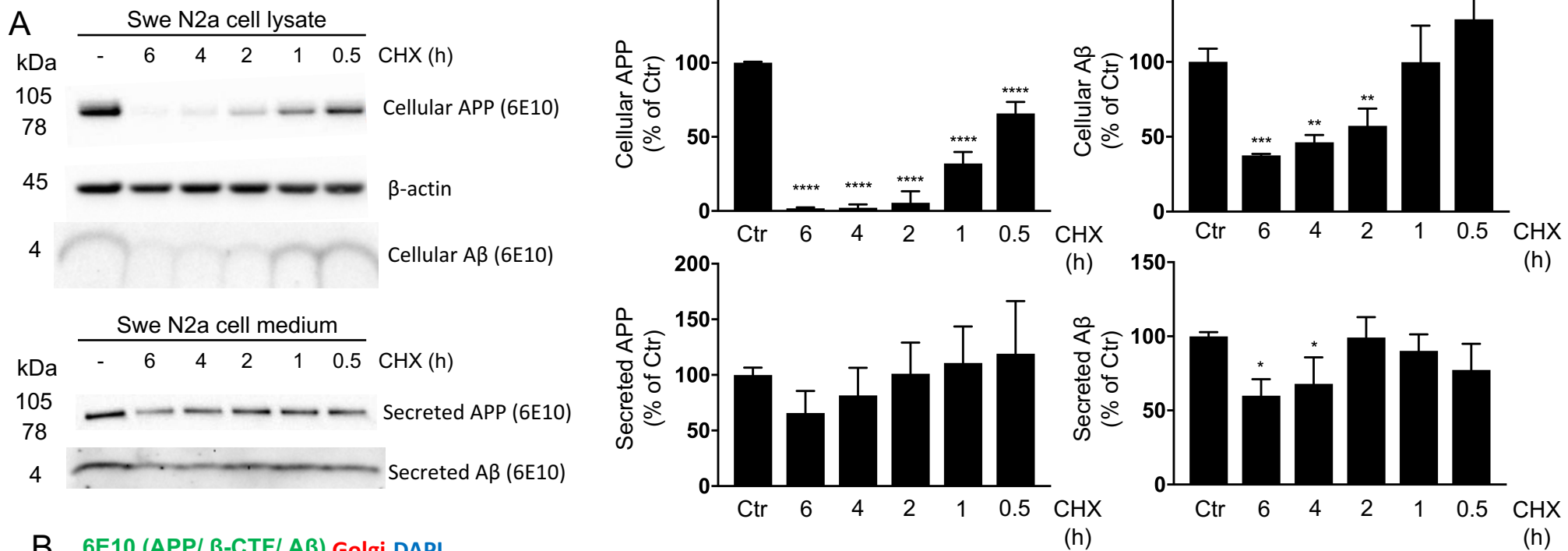

**B** **6E10 (APP/  $\beta$ -CTF/ A $\beta$ ) Golgi DAPI**

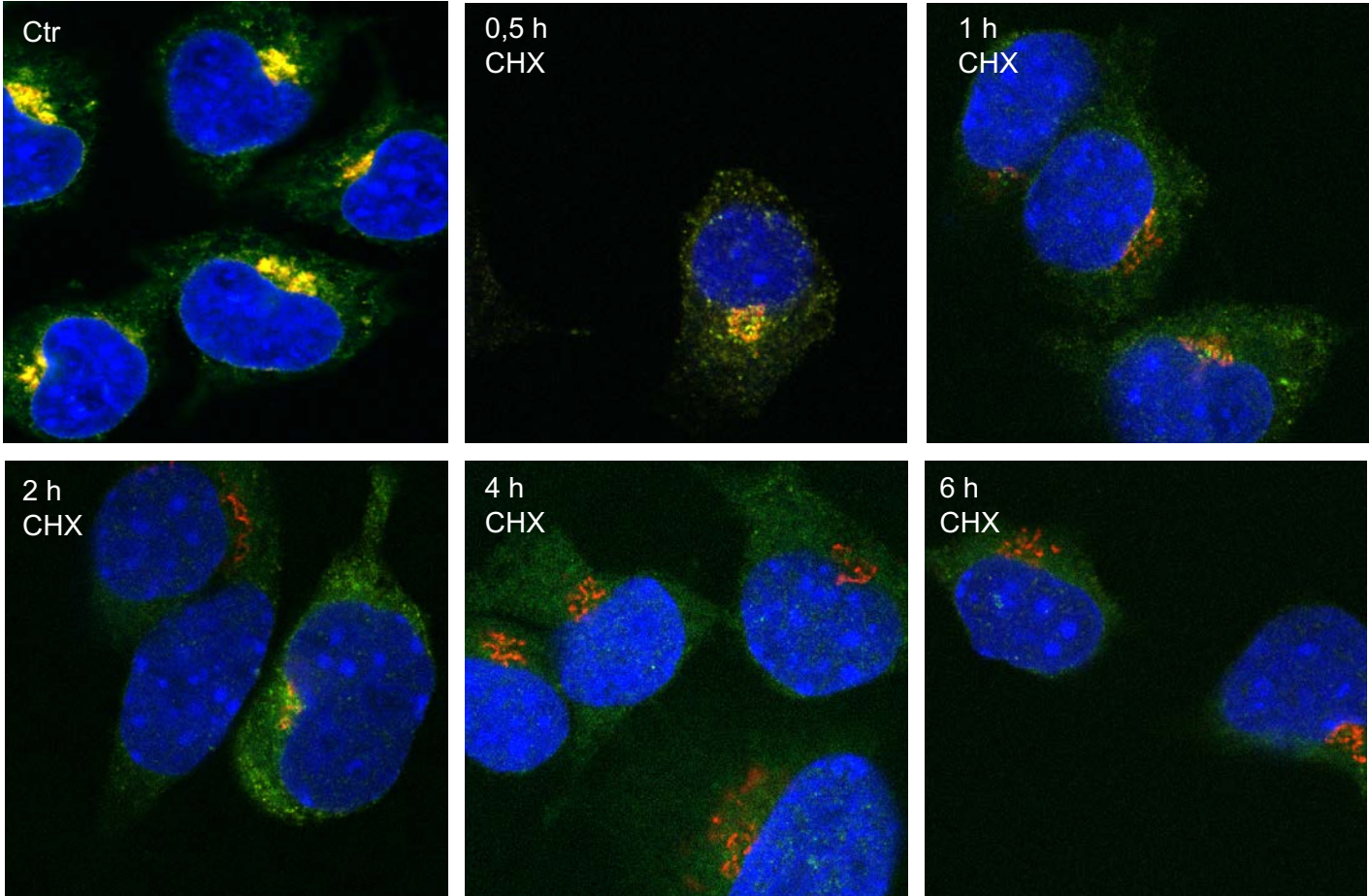

**6E10 (APP/  $\beta$ -CTF/ A $\beta$ )**

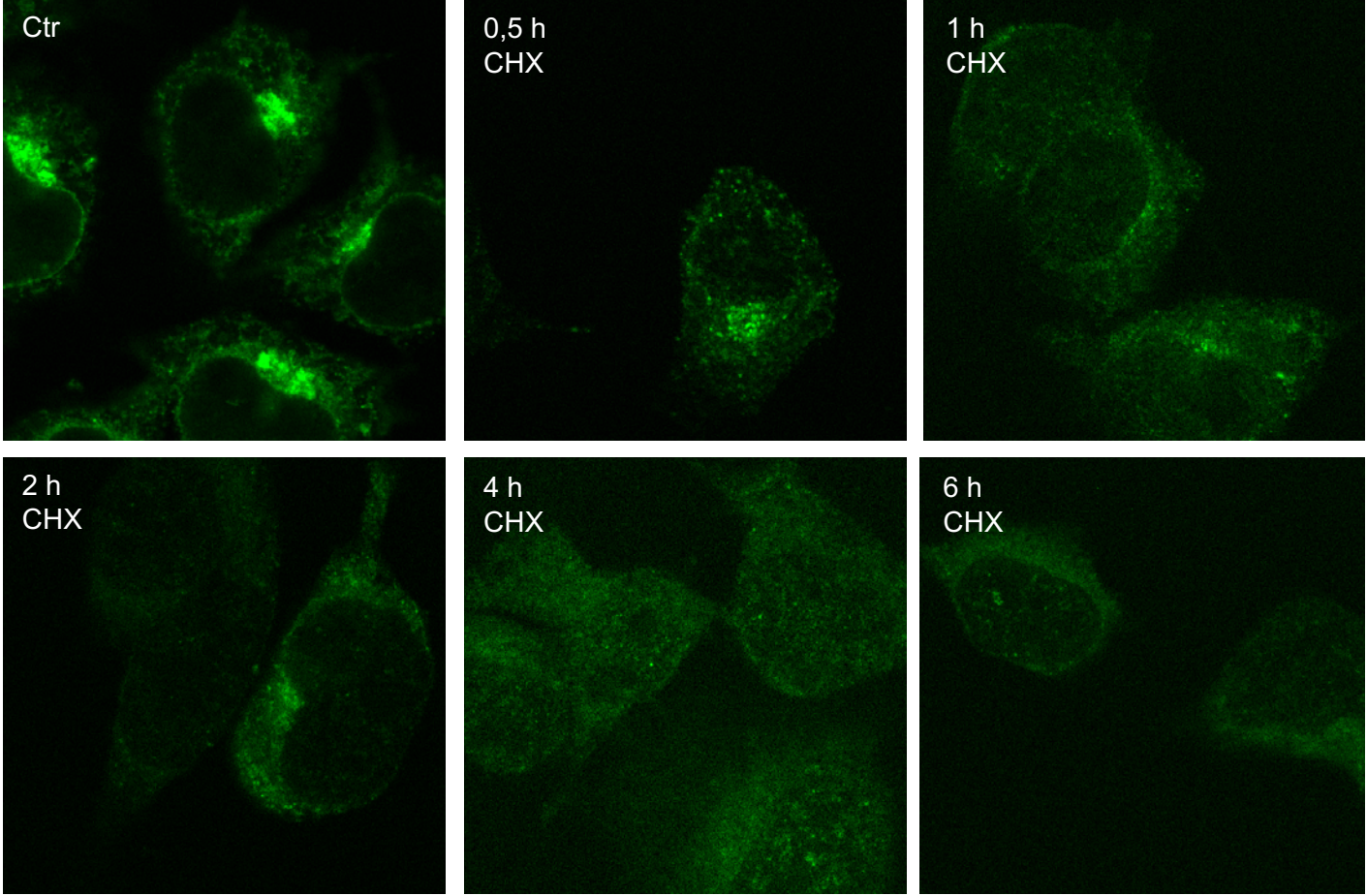

Supplement: Supplementary file 9 — (A) Western blot analysis of APP and Aβ in Swe N2a cells treated with 5 nM bafilomycin A1 (BafA1) at different time points (h) before harvest. Cell culture media was replaced with fresh media 24 h before harvest. (B) Quantification of A. Values are normalized against actin and expressed as percentage of control, n = 3; *p < 0.05, **p < 0.01, ***p < 0.001, ****p < 0.0001 (ANOVA with Dunnett’s multiple comparisons test, compared to ctr). (C) Confocal images of 6E10 and LAMP1 labelling in Swe N2a cells treated with 5 nM bafilomycin A1 for the depicted times. At 24 h there is a build up of both 6E10 labelling and punctate LAMP1-positive structures. (D) Western blot analysis of APP and Aβ in Swe N2a cells treated with 5 nM bafilomycin A1 (BafA1) 24 h before harvest and 40 μg/ml cycloheximide (CHX) at different time points (h) before harvest. Cell culture media was replaced with fresh media 24 h before harvest, before the addition of Baf A1. (PDF 2264 kb) [file 13024_2017_203_MOESM8_ESM.pdf]

Figure S8

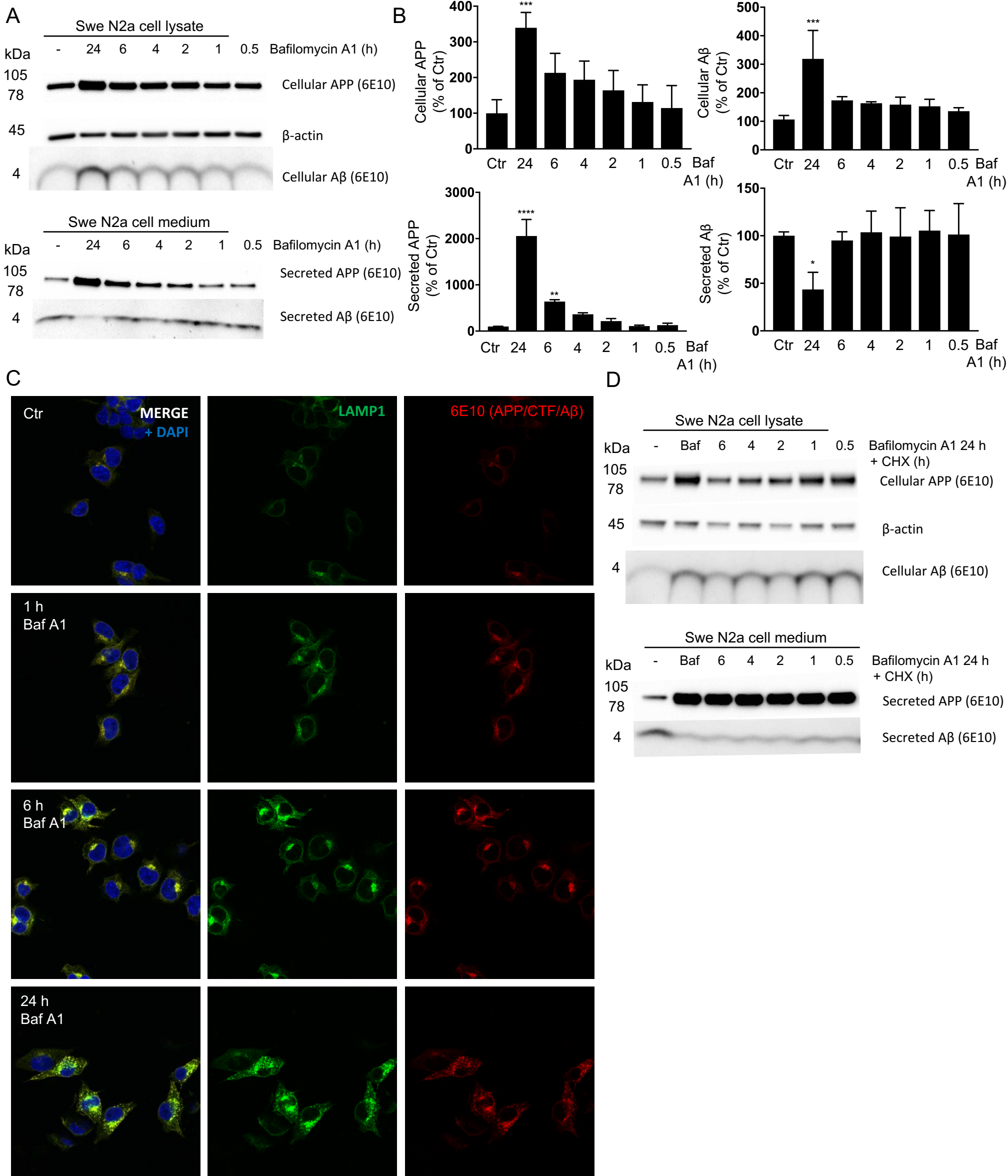

Supplement: Supplementary file 10 — (A) 3D images show increased GSK3β labelling in dnVPS4-expressing N2a Swe compared to cells transfected with control plasmid. Rab7 labelling is also increased in dnVPS4 expressing cells. Scale bar 15 μm. (B) Western blot analysis of cell lysates of Swe N2a cells transfected with dnVPS4A showing no changes in total GSK3β or phosphorylated GSK3α/β (serine 21/9). (PDF 1315 kb) [file 13024_2017_203_MOESM9_ESM.pdf]
