# Supplementary material for: Aβ accumulation causes MVB enlargement and is modelled by dominant negative VPS4A
Source: Mol Neurodegener. 2017 Aug 23;12:61. doi: 10.1186/s13024-017-0203-y (PMC5569475; doi:10.1186/s13024-017-0203-y)
Supplement: Supplementary file 2 — (A) EM image of an enlarged MVB in wt neurons treated with Aβ1-42. This image shows an example of an enlarged MVB with very few ILVs. (B) Confocal analysis of changes in size of LAMP1-positive structures in Aβ1-42 treated wt primary neurons with time. Scale bar 40 μm. (PDF 34 kb) [file 13024_2017_203_MOESM1_ESM.pdf]

Table S1

Antibody	Target Epitope	Species and type	Dilution (WB)	Dilution (IF)	Source	Cat. #
369	Human/mouse full length APP, α/β CTFs, APP C-terminus	Rabbit polyclonal	1:1000		Buxbaum et al. 1990 [29]	
6E10	Human Aβ, full length APP, sAPPα, β CTF, a.a. 3-8 of Aβ	Mouse monoclonal	1:1000	1:500	BioLegend	previously Covance SIG-39320
12F4	Human/mouse Aβx-42, C-terminus specific	Mouse monoclonal		1:250	BioLegend	Previously Covance SIG-39142
Amyloidβ (1-42)		Rabbit polyclonal		1:250	IBL	18582
Amyloidβ (1-42)	Human/mouse Aβx-42, C-terminus specific	Rabbit polyclonal		1:1000	Invitrogen	700254
β-actin		Mouse monoclonal	1:2000		Sigma	A 5316
CD63		Mouse monoclonal	1:1000		ThermoFisher Scientific	MAI-19281
CHMP2B		Rabbit polyclonal	1:1000	1:250	Abcam	ab33174
Clavestin-1+2		Rabbit polyclonal		1:250	Bioss	bs-6569R-A647
DAPI				1:1500	Sigma	D9542
Drebrin		Rabbit polyclonal		1:1000	Abcam	ab11068
FLAG	DYKDDDDK	Rat	1:1000	1:1000	Biolegend	637302
Flotillin-1		Mouse monoclonal		1:400	BD Biosciences	610821
GM130		Mouse monoclonal		1:500	BD Biosciences	610822
GSK3β		Rabbit monoclonal	1:1000	1:400	Cell Signaling Technology	12456
pGSKα/β		Rabbit polyclonal	1:1000		Cell Signaling Technology	9331S
HA-Tag		Rabbit monoclonal	1:1000		Cell Signaling Technology	3724
Hrs and Hrs-2		Mouse monoclonal		1:100	Enzo	ALX-804-382-C050
LAMP1		Rabbit polyclonal		1:1000	Abcam	ab24170
LAMP1		Rat monoclonal		1:1500	Abcam	ab25245
LC3β		Rabbit polyclonal	1:1000		Cell Signaling Technology	2775
OC	Amyloid fibrils	Rabbit polyclonal		1:1000	Merck Millipore	AB2286
P2:1	Human APP, N-terminus, a.a. 104-118 of APP	Mouse monoclonal		1:500	ThermoFisher Scientific	OMA1-03132
Phospho-tau pSer396		Rabbit polyclonal	1:1000		ThermoFisher	44-752G
Rab7		Mouse monoclonal		1:500	Abcam	ab50533
Synapto-physin		Mouse monoclonal		1:1000	Merck Millipore	MAB5258
Tsg101		Mouse monoclonal		1:250	Genetex	GTX70255
VPS4		Mouse monoclonal	1:1000	1:100	SantaCruz	sc-133122
